# “Help! I Need Somebody”: Music as a Global Resource for Obtaining Wellbeing Goals in Times of Crisis

**DOI:** 10.3389/fpsyg.2021.648013

**Published:** 2021-04-14

**Authors:** Roni Granot, Daniel H. Spitz, Boaz R. Cherki, Psyche Loui, Renee Timmers, Rebecca S. Schaefer, Jonna K. Vuoskoski, Ruth-Nayibe Cárdenas-Soler, João F. Soares-Quadros, Shen Li, Carlotta Lega, Stefania La Rocca, Isabel Cecilia Martínez, Matías Tanco, María Marchiano, Pastora Martínez-Castilla, Gabriela Pérez-Acosta, José Darío Martínez-Ezquerro, Isabel M. Gutiérrez-Blasco, Lily Jiménez-Dabdoub, Marijn Coers, John Melvin Treider, David M. Greenberg, Salomon Israel

**Affiliations:** ^1^Department of Musicology, The Hebrew University of Jerusalem, Jerusalem, Israel; ^2^Department of Psychology, The Hebrew University of Jerusalem, Jerusalem, Israel; ^3^The Jerusalem School of Business Administration, The Hebrew University of Jerusalem, Jerusalem, Israel; ^4^The Federmann Center for the Study of Rationality, The Hebrew University of Jerusalem, Jerusalem, Israel; ^5^Department of Music, College of Arts, Media and Design, Northeastern University, Boston, MA, United States; ^6^Department of Music, The University of Sheffield, Sheffield, United Kingdom; ^7^Health, Medical & Neuropsychology Unit, Institute of Psychology, Leiden University, Leiden, Netherlands; ^8^Academy for Creative and Performing Arts, Leiden University, Leiden, Netherlands; ^9^Centre for Interdisciplinary Studies in Rhythm, Time and Motion (RITMO), University of Oslo, Oslo, Norway; ^10^Escuela de Música, Facultad de Ciencias de la Educación, Universidad Pedagógica y Tecnológica de Colombia, Tunja, Colombia; ^11^Department of Music, Federal University of Ouro Preto, Ouro Preto, Brazil; ^12^Department of Psychology, Central China Normal University, Wuhan, China; ^13^Department of Psychology, University of Milano-Bicocca, Milan, Italy; ^14^Laboratory for the Study of Musical Experience, Facultad de Artes, Universidad Nacional de La Plata, La Plata, Argentina; ^15^Department of Developmental and Educational Psychology, Universidad Nacional de Educación a Distancia, Madrid, Spain; ^16^Facultad de Música, Universidad Nacional Autónoma de México, UNAM, Mexico City, Mexico; ^17^Unidad de Investigación Epidemiológica y en Servicios de Salud, Área Envejecimiento (UIESSAE), Centro Médico Nacional Siglo XXI, Instituto Mexicano del Seguro Social (IMSS) & Centro de Ciencias de la Complejidad (C3), Universidad Nacional Autónoma de México (UNAM), Mexico City, Mexico; ^18^Independent Researcher, Málaga, Spain; ^19^Laboratory of Psychology and Musical Arts, Faculty of Psychology and Faculty of Music, National Autonomous University of Mexico (UNAM), Mexico City, Mexico; ^20^Interdisciplinary Department of Social Sciences and Department of Music, Bar-Ilan University, Ramat Gan, Israel; ^21^Department of Psychiatry, Autism Research Centre, University of Cambridge, Cambridge, United Kingdom

**Keywords:** music, COVID-19, wellbeing, individualistic and collectivistic cultures, mood regulation, nostalgia, gender, age

## Abstract

Music can reduce stress and anxiety, enhance positive mood, and facilitate social bonding. However, little is known about the role of music and related personal or cultural (individualistic vs. collectivistic) variables in maintaining wellbeing during times of stress and social isolation as imposed by the COVID-19 crisis. In an online questionnaire, administered in 11 countries (Argentina, Brazil, China, Colombia, Italy, Mexico, the Netherlands, Norway, Spain, the UK, and USA, *N* = 5,619), participants rated the relevance of wellbeing goals during the pandemic, and the effectiveness of different activities in obtaining these goals. Music was found to be the most effective activity for three out of five wellbeing goals: enjoyment, venting negative emotions, and self-connection. For diversion, music was equally good as entertainment, while it was second best to create a sense of togetherness, after socialization. This result was evident across different countries and gender, with minor effects of age on specific goals, and a clear effect of the importance of music in people's lives. Cultural effects were generally small and surfaced mainly in the use of music to obtain a sense of togetherness. Interestingly, culture moderated the use of negatively valenced and nostalgic music for those higher in distress.

## Introduction

Music is a powerful stimulus that can drive our affective states, express complex and sometimes contradictory emotions, and energize or calm us (Juslin and Västfjäll, 2008; Juslin and Sloboda, 2010). Numerous studies have shown that music has profound influences on our perception (e.g., Bhattacharya and Lindsen, 2016), behavior (e.g., Schwartz et al., 2017), physiology (e.g., Grewe et al., 2007a,b), endocrine responses (e.g., Fancourt et al., 2014), and brain activity—especially in the emotional circuits of the brain and its associated reward system (Koelsch, 2014; Gold et al., 2019). It is therefore not surprising that mood regulation—venting negative emotions, maintaining positive mood, immersion in heightened emotion, energizing or relaxing—is repeatedly cited as one of the most important reasons for consuming music (Lonsdale and North, 2011; Baltazar and Saarikallio, 2016). Music listening also serves multiple goals or objectives with regard to wellbeing. These goals include aesthetic enjoyment, socializing, relieving loneliness, defining self-identity, recalling autobiographical memories, relieving boredom or unpleasant silences, and as a background to obtain optimal mental or physical performance. These goals may, in turn, change across the developmental trajectory, and vary across genders, cultures, personalities, and levels of musical training.

While all of these goals seem to be important and beneficial to our mental health and wellbeing, they may be even more important under conditions of high stress and social distancing as imposed by the COVID-19 pandemic. Alternatively, they may be felt as less relevant under these extreme conditions, or other activities may fare better in obtaining all or some of these goals.

The world has known extreme crises over the past 100 years. Although we cannot yet fully evaluate the severity of the current crisis in terms of its health, economic, social, and political outcomes, it clearly is one of the most severe ones we have known. The pandemic has affected each and every citizen directly: many feel that their health and even life is directly threatened, their daily life disrupted, their economic situation under threat, and their social support limited to distant electronic communication. Lockdown—limiting mobility to a restricted set of activities, or applying restrictions to work, school, leisure activities, tourism, and social gatherings –has taken its toll on wellbeing and mental health, with levels of stress, anxiety, depression, loneliness, and violence within families, on the rise in many countries (Banerjee and Rai, 2020; Holmes et al., 2020; Shader, 2020; Usher et al., 2020).

In a sense, this situation has created a natural experiment, in which the stressor is relatively comparable (though varying in severity, breadth, and exact timing), and the effects of daily activities—specifically the use of music—can be measured at the same time in all countries. In contrast with world crises in previous decades, technological advances have made music listening accessible to a vast majority of the world population. Therefore, the pandemic offers a unique opportunity to examine the importance of music in people's lives, and its ability to reduce stress, anxiety, and loneliness cross-culturally. The current research aims to examine the ability of music, in comparison to a wide array of other activities, to achieve goals for wellbeing during the COVID-19 pandemic. This aim is accomplished through a web-based questionnaire administered in six languages across 11 different countries: Argentina, Brazil, China, Colombia, Italy, Mexico, the Netherlands, Norway, Spain, the UK, and USA, to which 6,451 participants responded in total.

Only a small number of studies have examined the role of music and its uses in a cross-cultural perspective (Juslin et al., 2016). This paucity of studies is surprising given that music is both a social and cultural activity, and as such may reflect the values, norms, and identities of a given society. Differences in the use of music to support wellbeing under stressful circumstances could stem from variations in demographics, trust in the state as institution and living circumstances (Oksanen et al., 2020), prioritization of wellbeing and emotion objectives (Kitayama et al., 2000; Lim, 2016), the way emotions and negative events are processed (Miyamoto et al., 2014), and differences in the relative weight of the individual as an independent, self- sufficient, achievement-oriented agent (Individualistic Cultures), and societies in which individuals view themselves as interdependent, family and community centered (Collectivistic Cultures).

A number of studies found, overall, cross-cultural similarity in the use of music in daily life (Rana and North, 2007; Schäfer et al., 2012) or its use to obtain mood regulation and wellbeing goals (Saarikallio, 2008; Boer and Fischer, 2012). These studies also note some culture-dependent characteristics. For example, Boer et al. (2012) highlight differences in music use for socio-cultural functions of national and family identity—partially explained by the Individualistic-Collectivistic dimension (Boer and Abubakar, 2014). Similarly, Saarikallio et al. (2020) point to differences between Finns and Indians in functions of music with self-related functions (self-expression, self-enhancement, self-reflective) rated highly by Finns, and mood management goals (relaxation, motivation) more prevalent among Indians. The cultural dimension of Individualistic-Collectivistic was also significant in Juslin et al. (2016). They found many cross-cultural similarities in the prevalence of the emotional reactions to music, their underlying psychological mechanisms, and reasons for listening to music, yet they also found some differences. For example, individualistic listeners reported more negative affect elicited by music (e.g., sadness-melancholy), while collectivistic individuals were more prone to feel higher levels of nostalgia, transcendence, and happiness.

### Music vs. Other Activities

Music is one of several activities that people may employ to alleviate stress and anxiety and to boost their emotional and mental wellbeing, as also discussed above. As a leisure activity, it has many comparatives, ranging from organized sports to arts and craft, as well as to home activities such as cooking, gardening, and reading. It has been argued that music may be particularly effective to support emotional wellbeing, as it offers a means to express and regulate emotions (Laukka, 2007; Saarikallio, 2011), communicate non-verbally, connect with oneself, and others (Schäfer et al., 2013), and to be physically as well as mentally engaging, through dancing, singing, or playing. Systematic comparisons between the efficacy of musical vs. other forms of occupational interventions on various outcome measures, have been conducted in clinical and developmental settings primarily and are generally made between a small number of activities, such as participating in an arts or music class, a cooking or music group, a sport or music activity (e.g., Moreno et al., 2009; Narme et al., 2014; Alessandri et al., 2020). In the case of our research, we are comparing music as a coping strategy or rather a “coping activity” in comparison to other activities. From research on uses of music in everyday life, we know that everyday episodes that include music are more often associated with positive emotions, as well as experiences of nostalgia, than everyday life episodes without music (Juslin and Laukka, 2004). However, these studies did not compare across different types of positive, self-chosen everyday activities. As far as we are aware, few studies have made such a systematic comparison. One such study compared music with other media activities such as computer games, TV, Radio, Films, Newspaper, Sports, books, favorite hobbies (Lonsdale and North, 2011). They reported that mood management—both positive and negative—was significantly higher in the music activity than the other activities. Self-identity was highest in the music activity and hobbies, whereas diversion and release from boredom was mostly obtained through music and TV. In the current study, we extend significantly the range of activities with a deliberate attempt to include a range of comparative activities, that vary in degree of activation (reading—exercise), creativity (seeking information—arts and craft), and utility (entertainment—doing work or home improvements).

Given the unique situation of a global crisis and the paucity of studies on cross-cultural uses of music and other activities to address wellbeing goals, we distributed a survey to 11 countries, 5 of which can be classified as collectivistic and 6 as individualistic cultures. Participants were asked to indicate the importance of different wellbeing goals during lockdown. Subsequently for each wellbeing goal that was at least of some importance, they were asked to evaluate the degree to which various activities including music had been effective to address the goal. This was followed by specific questions on music consumption during the crisis. Furthermore, information was collected on demographics, personality, depression, stress, and anxiety. Together the survey enables us to investigate uses of music to support wellbeing, how this relates to the effectiveness of other home activities, how this is similar or variable across cultures, as well as dependent on personality, age, and proneness to depression and anxiety.

## Method

### Participants

The sample was drawn from 11 countries: Argentina, Brazil, China, Colombia, Italy, Mexico, the Netherlands, Norway, Spain, the UK, and USA. To enable the aggregation of data across countries for higher statistical power, we used Hofstede's (2001) broad conceptual framework of individualism vs. collectivism to categorize the 11 countries into 6 “individualistic” and 5 “collectivistic” countries. The individualistic countries were Anglophone and non-Anglophone Western countries (Italy, The Netherlands, Norway, Spain, UK, and USA). The collectivistic countries were South American and Asian (Argentina, Brazil, China, Colombia, and Mexico). Selection of countries was based on severity of the pandemic in the first wave (hence African countries were less relevant), and on willingness of collaborators to be involved in the project.

Out of 6,451 total participants that filled out our questionnaire, 5,619 were used for analysis after removing responses that were incomplete (below 95%) or that were not from one of the 11 targeted countries. Participants were recruited via academic websites (prolific.co), advertisements on the internet through social media, mailing lists, and universities' home page. Therefore, this is a convenience rather than a representative sample (Visser et al., 2000).

Participation was anonymous and participants received course credit or little to no compensation for completing the survey. Upon completing the survey, responders were shown their scores on a Big-5 questionnaire and were debriefed on the study. The study received IRB approval from the Hebrew University of Jerusalem Israel—the home university of the research leader (Roni Granot) in whose country (Israel) a big pilot study was run in order to optimize the study design. In the Netherlands, the local ethical committee of the Leiden University Psychology institute approved the study under application number V1-2549.

Supplementary Table 1 summarizes the demographic characteristics of the sample in terms of age, gender, experience of playing musical instruments, participation in balcony singing, distribution of platforms for music listening, experience with using electronic platforms for joint music singing or playing, depression, anxiety, stress (DASS21; Lovibond and Lovibond, 1995), and resilience (CD-RISC; Connor and Davidson, 2003) scores. This Table is followed by Appendix 1 in the Supplementary Materials describing, in general, the situation in each of the countries participating in the study in terms of severity of the pandemic and measures taken to control it during the months of data collection highlighting the fact that despite the similarity in the nature of the stressor there are also variations in severity and coping measures.

### Materials and Procedure

An online questionnaire was created on the Qualtrics platform and administered between July and November 2020 in six languages (English, Spanish, Dutch, Chinese, Italian, and Norwegian). Translations were done by the participating researchers, who were native speakers from each country. Spanish versions were adjusted for language specificities in each Spanish speaking country. Responses were saved on Qualtrics's server and then downloaded for analysis.

Before answering the questionnaire, a brief cover letter appeared on the first page, prior to the decision whether to take part in the study. It was explained that data collected would be used only for research purposes and that the participant was free to withdraw from the study at any time. The average duration needed to fill out the survey was ~15 min. The introduction section explained that “The purpose of this research is to gain a better understanding of how different people cope with the COVID19 crisis by using daily activities, especially music, in order to reduce negative feelings and maintain wellbeing in these complex times.” This wording was deemed by us as a reasonable compromise between reflecting the content of the questionnaire and reducing (though possibly not totally removing—see also caveats) bias of those responding to it.

The questionnaire included 7 sections of which the first 2 were **“**Goals” and **“**Activities.” These two sections which were at the core of the study, were set to always appear first, and the section on demographics and worry about COVID-19, as well as some country-specific questions which are not taken into account here, always appeared last. Other sections appeared in a randomized order. Items within non-standardized scales appeared also in a different randomized order per participant. One section which included non-standardized questions regarding beliefs and feelings provoked by the crisis such as “I feel lonely,” “I long for the times before the crisis,” **“**I am worried about the situation in my country,” did not result in a stable structure and was omitted from further analyses.

1. Goals:
  Participants were asked to rank how important on a five-point Likert scale (from 0 “irrelevant” to 4 “very large degree”) were five wellbeing goals in their coping with the lockdown and the situation that was imposed on them.
  a) Release and venting of negative emotions (e.g., stress, anxiety, anger).
  b) Diversion from the crisis.
  c) Enjoyment and maintaining good mood.
  d) Reducing loneliness and creating a sense of togetherness.
  e) Connecting with myself and detachment from the surroundings.
  f) Other (please indicate what in the blank space).
2. Activities:
  Goals receiving the importance level of two (“some degree”) or above, were followed by an Activities section, in which for each goal participants were asked to indicate how 10 activities contributed or interfered with attaining the specific goal. They scored each activity on a seven-point Likert scale, ranging from “significantly prevented” to “significantly helped” (with a neutral option in the middle for cases where activity was either irrelevant (not used at all) or did not help nor prevent). The activities were:
  a) Information seeking (news on the TV or internet).
  b) Entertainment (e.g., movies, electronic games, series).
  c) Music (e.g., listening, playing an instrument, singing).
  d) Food (i.e., eating or cooking).
  e) Physical activity (e.g., walking, exercise, dance).
  f) Doing productive activities (e.g., cleaning, renovating, work).
  g) Reading or listening to books, magazines, or podcasts.
  h) Talking or socializing with others (via zoom, phone, face to face).
  i) Engaging with things I like (e.g., hobbies, pets).
  j) Spirituality and mindfulness (e.g., praying, meditation).
  After finishing this task, each participant completed the next blocks in a randomized order, besides the demographic questionnaire that always appeared at the end of the study.
3. Personality Traits:
  *Ten-Item Personality Inventory (TIPI)*: A brief version of the Big Five Inventory (Costa and McCrae, 1992), which measures Extraversion, Agreeableness, Conscientiousness, Emotional Stability (Neuroticism), and Openness to Experience, each with two items per trait (Gosling et al., 2003). Psychometric tests of TIPI show adequate levels in terms of convergence with widely used Big Five measures in self, observer and peer reports, test-retest reliability, patterns of predicted and external correlates (Gosling et al., 2003). The dimensions of the “Big Five” model have also been identified in non-Western societies (Church and Katigbak, 1989), and are assumed to be enduring and “biologically anchored” dispositions (Allik and McCrae, 2002). The Chinese (Carciofo et al., 2016), Dutch (Hofmans et al., 2008), Italian (Chiorri et al., 2015), Portuguese (Nunes et al., 2018), and Spanish (Romero et al., 2012; Ruiz et al., 2013), versions were validated for their respective samples.
4. Emotional State:
  *The Depression, Anxiety, Stress Scale (DASS-21)*: A 21 items self-report tool that assesses depression, anxiety and stress using 7 items for each scale (Lovibond and Lovibond, 1995). Participants are asked to read statements (e.g., “I found it hard to wind down”) and to state how much these sentences applied to them over the past week using a 4-point Likert-type scale ranging from 0 (“Did not apply to me at all”) to 3 (“Applied to me very much or most of the time”). The results of each scale are calculated by summing the 7 items that comprise the respective scales. The questionnaire is widely employed in both research and clinical assessment and is available in numerous translations studied and approved for validation and reliability as well as factorial structure (e.g., Mellor et al., 2015; Zanon et al., 2020).
5. Resilience:
  *Connor-Davidson Resilience Scale (CD-RISC)*: The scale was developed by Connor and Davidson (2003) for clinical screening as assessment of mental health as well as for evaluating treatment effectiveness. Respondents rate items on a scale from 0 (not true at all) to 4 (true nearly all the time) based on how they felt over the past month. For this study we used the 10-items version (Campbell-Sills and Stein, 2007) which ranges from 0 to 40 with higher scores indicating higher resilience. There are no existing norms in the literature for culture, gender, or age groups, as resilience is considered situation dependent.
6. Music Use During the Crisis:
  The block was composed of a series of different questions focused on music use, consumption and participation during lockdown as detailed in Appendix 2 in the Supplementary Materials. These questions asked participants to indicate how important music is for them in general, how much time, they estimated, was spent on music listening during the lockdown as compared to prior to lockdown, and characteristics of music listened to in terms of emotional valence and arousal, nostalgia, and language. Participants were asked whether they had joined balcony singing or hand clapping, and what the effect of these were on their mood, and a sense of togetherness with their community (those not participating in these activities were also asked about their sense of togetherness with the community). Finally, participants indicated channels of consuming music, online engagement with various cultural activities and provided examples of songs that helped them cope with the situation (these last items were not analyzed in the current study).
7. Demographics and Worry about COVID-19:
  The demographic part of the questionnaire (slightly adjusted per country) included questions regarding, age, gender, first and other languages, ethnicity, religion and level of religiosity, relationship status, general education, musical education, where and with whom did the participants pass the lockdown, and experience and worry about the COVID-19 (having been tested, having symptoms, being at risk, knowing someone who died) and scoring of six statements in response to the question “to what degree are you worried about the following…?” on a five-point Likert scale, ranging from 0 “not at all” to 4 “extremely”:
  a) Getting COVID-19.
  b) Dying from COVID-19.
  c) Family members or close friends getting COVID-19.
  d) Unknowingly infecting others with COVID-19.
  e) Currently having COVID-19 (even though you're pretty sure you don't).
  f) Having significant financial burden because of the COVID-19 pandemic.

## Results

As shown in Supplementary Table 1, the number of respondents per country ranged from 177 (Norway) to 901 (Colombia) with a median of 495. On average, two thirds of the respondents were female (range among countries 52–73%). Respondents' ages ranged from 18 to over 64, with the older age group of over-64 being underrepresented (an average of 5.6% of the sample). Musical training ranged from no or little (<3 years) training (mean across countries = 52%) to over 12 years (*M* = 19.2%). The main platforms for music consumption were free channels such as YouTube and streaming services which together cover ~85% of mediums for consumption (with varying proportions among different countries), with other means such as “own CD collections” (~8.5%) or TV and radio much less common. That is, the vast majority of music listened to, was self-selected.

### Goal Importance by Culture

To examine differences in goal importance, and potential effects of cultures, we applied a multilevel regression model with respondents and countries as random factors, and Culture, Goal, and Culture × Goal interaction as fixed factors. To extract respondents and countries ICC (Inter-Class Correlation), we first applied an unconditional model (i.e., a model without any predictor; see Table 1, model-0). Next, we added the Goal as a predictor (see Table 1, model-1) and applied a pairwise comparison between the goals. In the next step, we added to the model the Culture variable, and the Culture × Goal interaction (see Table 1, model-2, model-3). Finally, we compared the estimated marginal means of each goal's importance between cultures. The reference goal is Venting negative emotions.

**Table 1:** Overview of main statistical outcomes of multilevel regression models testing the effect of Goals, Culture (collectivism vs. individualism) and their interaction on evaluated importance.

	**Model-0**		**Model-1**		**Model-2**		**Model-3**	
	**Estimates**	**CI**	**Estimates**	**CI**	**Estimates**	**CI**	**Estimates**	**CI**
**Intercept**	3.39^***^	3.26 to 3.51	3.40^***^	3.27 to 3.53	3.43^***^	3.24 to 3.63	3.50^***^	3.31 to 3.70
**Goal**								
Diversion			−0.33^***^	−0.37 to −0.29	−0.33^***^	−0.37 to −0.29	−0.57^***^	−0.62 to −0.52
Enjoyment			0.51^***^	0.48 to 0.55	0.51^***^	0.48 to 0.55	0.47^***^	0.42 to 0.52
Togetherness			−0.10^***^	−0.13 to −0.06	−0.10^***^	−0.13 to −0.06	−0.18^***^	−0.23 to −0.13
Self-Connection			−0.15^***^	−0.19 to −0.12	−0.15^***^	−0.19 to −0.12	−0.14^***^	−0.19 to −0.09
**Culture dummy**					−0.06	−0.32 to 0.20	−0.21	−0.47 to 0.06
**Goal × Culture**								
Diversion × Culture							0.49^***^	0.41 to 0.56
Enjoyment × Culture							0.09^*^	0.02 to 0.17
Togetherness × Culture							0.18^***^	0.10 to 0.25
Self-Connection × Culture							−0.04	−0.11 to 0.03
**Random effects**								
σ^2^	1.06		0.96		0.96		0.95	
τ_00__respondent_	0.34		0.36		0.36		0.36	
_country_	0.04		0.04		0.05		0.05	
N _respondent_	5,605		5,605		5,605		5,605	
_country_	11		11		11		11	
ICC _respondent_	0.235							
_country_	0.030							
Observations	27,920		27,920		27,920		27,920	
Marginal *R*^2^ /Conditional *R*^2^	0.000/0.265		0.056/0.335		0.056/0.335		0.063/0.344	

*Culture dummy = 1 for individualistic culture, 0 for collectivistic culture. CI, Confidence Interval. Reference Goal is Venting negative emotions*.

* p < 0.05 and

*** *p < 0.001*.

Chi-square test for comparison between models, revealed that the main effect of Goal was significant [$X_{\left(4\right)}^{2}$ = 2233.62, *p* < 0.001; marginal *R*^2^ = 0.06 (“medium” effect-size)]. To calculate the effect-size of the differences between goals' importance, we used the equation of Westfall et al. (2014) for calculating Cohen's *d* in multilevel models:

$$d=\frac{Difference\;between\;the\;means}{\sqrt{varintercept_{part}\;+\;varintercept_{item}+varslope_{part}+varslope_{item}+var_{residual}}}$$

**Figure 1:**
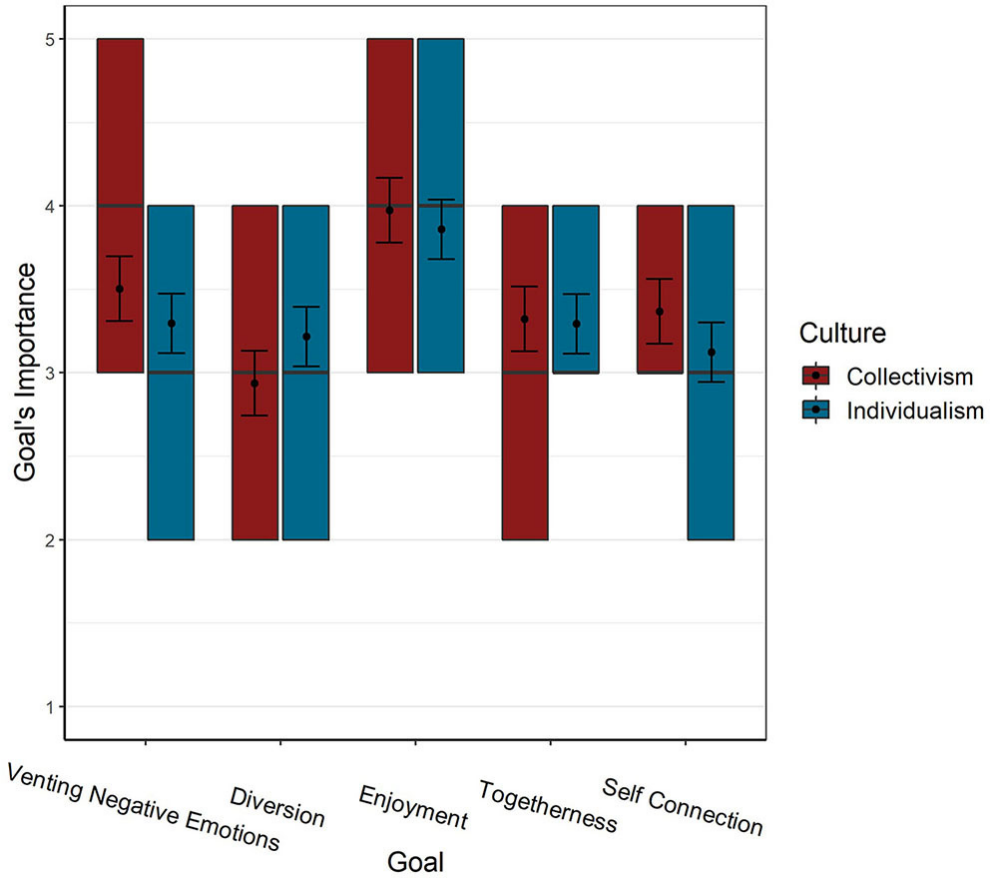
Importance of goals as a function of culture. Error bars represent 95% CI. Colored squares represent the 2nd and 3rd quartiles of responses. Black lines represent the median, and dots represent the mean.

As seen in Table 1 (“Estimates” in Model-1) and Figure 1, the most important goal was Enjoyment and maintaining a good mood (hereafter: Enjoyment), and the least important goal was Diversion from the Crisis (hereafter: Diversion). A *post-hoc* comparison between the goals' importance (with Bonferroni correction for multiple comparisons) shows that the contrast between the two is 0.84 (Cohen's *d* = 0.72; “medium size effect”). The contrast between Enjoyment and the other three goals: Connecting with myself and detachment from the surroundings (hereafter: Self-connection) (0.67), Reducing loneliness and creating a sense of togetherness (hereafter: Togetherness) (0.61), and Release and venting of negative emotions (hereafter: Venting) (0.51), shows a medium effect-size (Cohen's *d* = 0.57 and 0.52 for the first two, respectively); and a small effect-size (0.44) for Venting. All other differences are of a small or very small effect-size. The Culture × Goal interaction term was also a significant predictor of goals' importance [$X_{\left(4\right)}^{2}$ =254.96, *p* < 0.001], but as can be seen in Table 1, the marginal *R*^2^ difference between models with (Model-3) and without (Model-2) the interaction term is only 0.01 (“small” effect-size), indicating that, overall, the differences between goals' importance were quite similar across cultures. Note that the significant interactions (Model-3) indicate patterns of interaction between Goal and Culture different from those found between Venting negative emotions and Culture (e.g., for Togetherness a smaller difference between cultures as compared to Venting). This does not indicate if Cultures were significantly different from each other.

In order to examine these differences we used pairwise contrasts (see also Figure 1) which indicated that cultures differed only in the importance of Diversion [*p* = 0.022, Cohen's *d* = 0.24 (“small” effect-size)] and Self-connection [*p* = 0.045, Cohen's *d* = −0.15 (“very small” effect-size)]. Diversion was the only goal which received higher ratings in individualistic compared to collectivistic cultures.

### Music Efficiency Across Goals and Cultures

**Figure 2:**
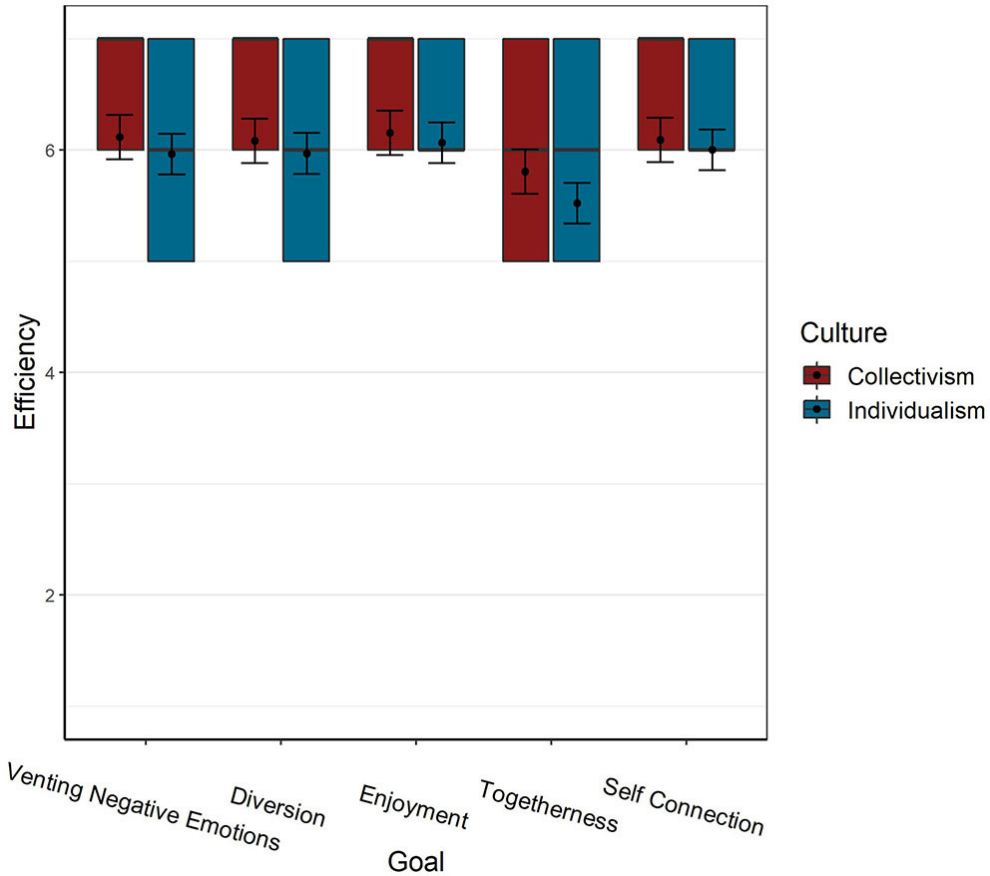
Efficiency of music in obtaining wellbeing goals by culture. Error bars represent 95% CI. Colored squares represent the 2nd and 3rd quartiles of responses. Black lines represent the median, and dots represent the mean.

To examine whether music was equally successful in obtaining the different goals and if this varied across cultures, we applied a multilevel regression model with respondents and countries as random factors, and Culture, Goal, and Culture × Goal interaction as fixed factors. To extract respondents and countries ICC, we first applied an unconditional model (i.e., a model without any predictor; see Table 2, model-0). Next, we added the Goal as a predictor (see Table 2, model-1) and applied a pairwise comparison between the music ratings per each of the goals with the rating of music in obtaining the goal of Venting negative emotions as reference. In the next step, we added to the model the Culture variable, and the Culture × Goal interaction (see Table 2, model-2, model-3). Finally, we compared the estimated marginal means of each music rating per goal between cultures (see also Figure 2).

**Table 2:** Overview of main statistical outcomes of multilevel regression models testing the efficiency of music in obtaining wellbeing goals by Goal and Culture.

	**Model-0**		**Model-1**		**Model-2**		**Model-3**	
	**Estimates**	**CI**	**Estimates**	**CI**	**Estimates**	**CI**	**Estimates**	**CI**
**Intercept**	5.98^***^	5.85 to 6.11	6.03^***^	5.90 to 6.17	6.11^***^	5.91 to 6.31	6.12^***^	5.92 to 6.32
**Goal**								
Diversion			−0.01	−0.04 to 0.02	−0.01	−0.04 to 0.02	−0.03	−0.08 to 0.01
Enjoyment			0.07^***^	0.04 to 0.10	0.07^***^	0.04 to 0.10	0.04	−0.00 to 0.08
Togetherness			−0.38^***^	−0.41 to −0.34	−0.38^***^	−0.41 to −0.34	−0.31^***^	−0.35 to −0.26
Self-Connection			0.01	−0.03 to 0.04	0.01	−0.03 to 0.04	−0.02	−0.07 to 0.02
**Culture dummy**					−0.14	−0.41 to 0.13	−0.15	−0.42 to 0.12
**Goal × Culture**								
Diversion × Culture							0.04	−0.03 to 0.10
Enjoyment × Culture							0.06^*^	0.00 to 0.12
Togetherness × Culture							−0.13^***^	−0.20 to −0.07
Self-Connection × Culture							0.06	−0.00 to 0.13
**Random effects**								
σ^2^	0.52		0.49		0.49		0.49	
τ_00__respondent_	0.76		0.77		0.77 _num_		0.77 _num_	
_country_	0.05		0.05		0.05 _country_		0.05 _country_	
N _respondent_	5,470		5,470		5,470 _num_		5,470 _num_	
_country_	11		11		11 _country_		11 _country_	
ICC _respondent_	0.569							
_country_	0.037							
Observations	20,512		20,512		20,512		20,512	
Marginal *R*^2^/Conditional *R*^2^	0.000/0.606		0.019/0.633		0.023/0.634		0.024/0.635	

*Culture dummy = 1 for individualistic culture, 0 for collectivistic culture. CI, Confidence Interval. Reference Goal is venting negative emotions*.

* p < 0.05 and

*** *p < 0.001*.

A Chi-square test indicated that music's efficiency was goal-dependent [$X_{\left(4\right)}^{2}$ = 969.33, *p* < 0.001]. However, as can be seen in Table 2, the *R*^2^ difference between the unconditional model (model-0) to the model with the Goal as predictor, is only 0.02 (“small effect-size”). Most of this effect was carried by the difference between the lower efficiency of music in obtaining Togetherness vs. all other goals which received higher ratings, similar to each other. Comparisons between music's efficiency across goals (with a Bonferroni correction for multiple comparisons) show that the largest contrast (0.45) is found between Enjoyment, which received the highest rating, and Togetherness (Cohen's *d* = 0.39: “small” effect-size). Similar effect sizes are found in the contrasts between music's rated effectiveness in achieving Togetherness as opposed to the remaining three goals: Venting, Self-connection (Cohen's *d* for both = 0.33 “small” effect-size) and Diversion (Cohen's *d* = 0.31 “small” effect-size). All other differences have a very small effect-size.

The Culture × Goal interaction term was also a significant predictor of music's efficiency [$X_{\left(4\right)}^{2}$ = 53.41, *p* < 0.001]. However, the marginal *R*^2^ difference between models with (Model-3) and without (Model-2) the interaction term is <0.01 (“very small”).

Pairwise contrasts (see Figure 2) indicated that cultures differed in music's efficiency only in Togetherness [*p* = 0.039, Cohen's *d* = −0.25 (“small”)], such that collectivistic cultures rated music's efficiency in obtaining this goal higher than individualistic ones.

### Music vs. Other Activities by Goal and Culture

**Figure 3:**
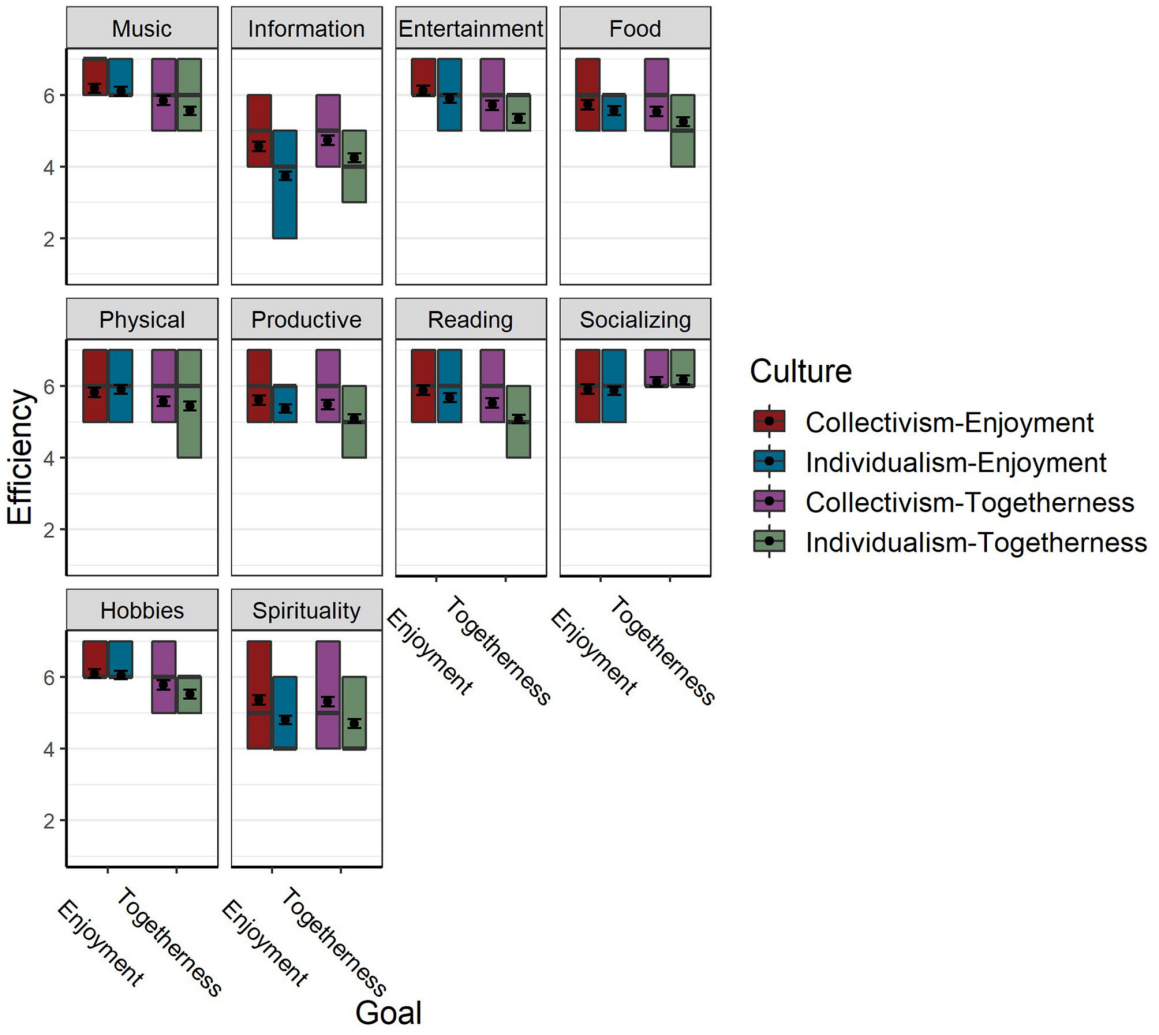
Efficiency of Activities in obtaining “Enjoyment and maintaining good mood” and “Reducing Loneliness and Creating a Sense of Togetherness” by culture. Colored squares represent the 2nd and 3rd quartiles of responses. Black lines represent the median, and “x” represents the mean.

Next, we asked whether the efficiency of music in obtaining the various goals differed from that of the other activities, and whether this was modulated by Culture. For each goal, we applied four multilevel regression models. Table 3 exemplifies the structure of these models: (1) unconditional model (model-0) with respondents and countries as random factors (for extracting ICC), (2) A model with Activity as fixed factor (the reference activity is Music; model-1), and models (3+4) with the Culture and the Activity × Culture interaction effects (model-2 and model-3). Here we present full tables only for the goal of obtaining Enjoyment (Table 3 and Figure 3), and the goal of Togetherness (Table 4 and Figure 3), for which music obtained highest and lowest ratings, respectively, among the five goals. Tables for the remaining goals are presented in the Supplementary Materials. Here we will only report results of the analyses.

**Table 3:** Overview of main statistical outcomes of multilevel regression models testing the efficiency of activities in obtaining “Enjoyment and maintaining good mood” by Activity and Culture.

	**Model-0**		**Model-1**		**Model-2**		**Model-3**	
	**Estimates**	**CI**	**Estimates**	**CI**	**Estimates**	**CI**	**Estimates**	**CI**
**Intercept**	5.60^***^	5.50 to 5.70	6.13^***^	6.03 to 6.24	6.25^***^	6.13 to 6.38	6.17^***^	6.04 to 6.30
**Activity**								
Information seeking			−2.00^***^	−2.04 to −1.95	−2.00^***^	−2.04 to −1.95	−1.62^***^	−1.68 to −1.55
Entertainment			−0.13^***^	−0.18 to −0.09	−0.13^***^	−0.18 to −0.09	−0.06	−0.12 to 0.00
Food			−0.50^***^	−0.55 to −0.46	−0.50^***^	−0.55 to −0.46	−0.46^***^	−0.52 to −0.39
Physical			−0.28^***^	−0.32 to −0.23	−0.28^***^	−0.32 to −0.23	−0.36^***^	−0.42 to −0.30
Productive			−0.66^***^	−0.70 to −0.61	−0.66^***^	−0.70 to −0.61	−0.57^***^	−0.64 to −0.51
Reading			−0.37^***^	−0.42 to −0.33	−0.37^***^	−0.42 to −0.33	−0.30^***^	−0.36 to −0.24
Socializing			−0.25^***^	−0.30 to −0.21	−0.25^***^	−0.30 to −0.21	−0.27^***^	−0.33 to −0.21
Hobbies			−0.07^**^	−0.11 to −0.02	−0.07^**^	−0.11 to −0.02	−0.09^**^	−0.15 to −0.02
Spirituality			−1.07^***^	−1.11 to −1.02	−1.07^***^	−1.11 to −1.02	−0.82^***^	−0.88 to −0.76
**Culture dummy**					−0.21^*^	−0.38 to −0.05	−0.06	−0.23 to 0.12
**Activity × Culture**								
Information × Culture							−0.75^***^	−0.84 to −0.67
Entertainment × Culture							−0.15^**^	−0.24 to −0.06
Food × Culture							−0.09^*^	−0.18 to −0.00
Physical × Culture							0.16^***^	0.07 to 0.25
Productive × Culture							−0.17^***^	−0.25 to −0.08
Reading × Culture							−0.14^**^	−0.23 to −0.05
Socializing × Culture							0.03	−0.06 to 0.12
Hobbies × Culture							0.03	−0.05 to 0.12
Spirituality × Culture							−0.49^***^	−0.57 to −0.40
**Random effects**								
σ^2^	1.63		1.26		1.26		1.24	
τ_00__respondent_	0.23		0.27		0.27		0.27	
_country_	0.03		0.03		0.02		0.02	
N _respondent_	4,907		4,907		4,907		4,907	
_country_	11		11		11		11	
ICC _respondent_	0.122							
_country_	0.015							
Observations	48,663		48,663		48,663		48,663	
Marginal *R*^2^/Conditional *R*^2^	0.000/0.137		0.174/0.330		0.180/0.330		0.188/0.340	

*Culture dummy = 1 for individualistic culture, 0 for collectivistic culture. CI, Confidence Interval. Reference Activity is Music*.

* *p < 0.05*,

** p < 0.01, and

*** *p < 0.001*.

**Table 4:** Overview of main statistical outcomes of multilevel regression models testing the efficiency of activities in “Reducing Loneliness and Creating a Sense of Togetherness” by Activity and Culture.

	**Model-0**		**Model-1**		**Model-2**		**Model-3**	
	**Estimates**	**CI**	**Estimates**	**CI**	**Estimates**	**CI**	**Estimates**	**CI**
**Intercept**	5.42^***^	5.29 to 5.55	5.72^***^	5.58 to 5.85	5.90^***^	5.77 to 6.03	5.88^***^	5.75 to 6.02
**Activity**								
Information seeking			−1.21^***^	−1.26 to −1.16	−1.21^***^	−1.26 to −1.16	−1.11^***^	−1.18 to −1.04
Entertainment			−0.18^***^	−0.22 to −0.13	−0.18^***^	−0.22 to −0.13	−0.14^***^	−0.20 to −0.07
Food			−0.31^***^	−0.36 to −0.26	−0.31^***^	−0.36 to −0.26	−0.31^***^	−0.38 to −0.25
Physical			−0.19^***^	−0.24 to −0.15	−0.19^***^	−0.24 to −0.15	−0.28^***^	−0.34 to −0.21
Productive			−0.41^***^	−0.46 to −0.36	−0.41^***^	−0.46 to −0.36	−0.36^***^	−0.43 to −0.30
Reading			−0.40^***^	−0.45 to −0.35	−0.40^***^	−0.45 to −0.35	−0.32^***^	−0.39 to −0.26
Socializing			0.44^***^	0.40 to 0.49	0.44^***^	0.40 to 0.49	0.27^***^	0.21 to 0.34
Hobbies			−0.05	−0.09 to 0.00	−0.05	−0.09 to 0.00	−0.06	−0.13 to 0.00
Spirituality			−0.69^***^	−0.74 to −0.64	−0.69^***^	−0.74 to −0.64	−0.53^***^	−0.60 to −0.46
**Culture dummy**					−0.33^***^	−0.50 to −0.16	−0.30^**^	−0.48 to −0.12
**Activity × Culture**								
Information × Culture							−0.20^***^	−0.29 to −0.11
Entertainment × Culture							−0.08	−0.17 to 0.01
Food × Culture							0.01	−0.09 to 0.10
Physical × Culture							0.17^***^	0.07 to 0.26
Productive × Culture							−0.10^*^	−0.20 to −0.01
Reading × Culture							−0.16^**^	−0.25 to −0.06
Socializing × Culture							0.34^***^	0.24 to 0.43
Hobbies × Culture							0.03	−0.06 to 0.13
Spirituality × Culture							−0.32^***^	−0.42 to −0.23
**Random effects**								
σ^2^	1.37		1.18		1.18		1.17	
τ_00__respondent_	0.41		0.43		0.43		0.43	
_country_	0.05		0.05		0.02		0.02	
N _respondent_	4,049		4,049		4,049		4,049	
_country_	11		11		11		11	
ICC _respondent_	0.223							
_country_	0.026							
Observations	40,142		40,142		40,142		40,142	
Marginal *R*^2^/Conditional *R*^2^	0.000/0.249		0.095/0.354		0.110/0.354		0.114/0.359	

*Culture dummy = 1 for individualistic culture, 0 for collectivistic culture. CI, Confidence Interval. Reference Activity is Music*.

* *p < 0.05*,

** p < 0.01, and

*** *p < 0.001*.

#### The Goals of Enjoyment and Maintaining Good Mood and Reducing Loneliness and Creating a Sense of “Togetherness”

The Chi-square test indicated that Activities were different in their efficiency [$X_{\left(9\right)}^{2}$ = 11080.85, *p* < 0.001] in obtaining Enjoyment and Togetherness [$X_{\left(9\right)}^{2}$ = 5467.58, *p* < 0.001]. The *R*^2^ difference between the unconditional model to the model with the activities effect is “large” for Enjoyment (0.17) and “medium” for Togetherness (0.10). As can be seen in Table 3 (model-1) and Figure 3, music was more efficient compared to all other activities in obtaining Enjoyment [*p* (Hobbies) = 0.002, all other *p*'s < 0.001, Cohen's *d* ranged from 0.05 (“very small”; Hobbies) to 1.59 (“large”; Information-seeking)]. As for Togetherness (Table 4 and Figure 3), music was more efficient compared to all other activities except for Socializing [which was more efficient than Music; *p* < 0.001, Cohen's *d* = −0.26 (“small”)], and Hobbies which was as efficient as Music (*p* > 0.05; all other *p's* < 0.001). Cohen's *d*'s ranged from 0.04 (“very small”; Hobbies) to 0.94 (“large”; Information-seeking). Culture by itself was also a significant predictor of activities' efficiency in obtaining Enjoyment [$X_{\left(1\right)}^{2}$ = 5.98, *p* = 0.014, marginal *R*^2^ = 0.01 (“small”)], and Togetherness [$X_{\left(1\right)}^{2}$ = 10.71, *p* = 0.001, marginal *R*^2^ = 0.02 (“small”)], with Collectivistic cultures rating activities overall higher than Individualistic cultures.

The Culture × Activity interaction term (see also Figure 3) was also a significant predictor of activities' efficiency [$X_{\left(9\right)}^{2}$ = 647.15, *p* < 0.001 and $X_{\left(9\right)}^{2}$ = 271.97, *p* < 0.001 for Enjoyment and Togetherness, respectively], but the marginal *R*^2^ difference between models with (Model-3) and without (Model-2) the interaction term is only 0.01 or smaller (“small-very small”). Cohen's *d*'s for differences in activities' efficiencies between cultures ranged from −0.65 (“medium”; Information-seeking) to 0.08 (“very small”; Physical activity) for Enjoyment, and −0.49 (“small”; Spirituality) to 0.03 (“very small”; Socializing) for Togetherness. Efficiency ratings tended to be higher for collectivist cultures with relatively large differences for Information seeking and Spirituality. The trend was the opposite for Physical activity, and very marginal differences for Hobbies and Socializing.

#### Venting Negative Emotions

Chi-square test indicated that activities were different in their efficiency in Venting negative emotions [$X_{\left(9\right)}^{2}$ = 8714.81, *p* < 0.001]. The marginal *R*^2^ difference between the unconditional model to the model with the activities effect is 0.16 (“large”). As can be seen in Supplementary Table 2 (model-1) and Supplementary Figure 1, music was more efficient compared to all other activities in Venting negative emotions [all *p*'s < 0.001, Cohen's *d*'s ranged from 0.14 (“very small”; Hobbies and Physical activity) to 1.60 (“large”; Information-seeking)].

The Culture × Activity interaction term was also a significant predictor of activities' efficiency [$X_{\left(9\right)}^{2}$ = 363.35, *p* < 0.001], but as can be seen in Supplementary Table 2, the marginal *R*^2^ difference between models with (Model-3) and without (Model-2) the interaction term is only 0.01 (“small”).

Cohen's *d*'s for differences in activities' efficiencies between cultures ranged from −0.57 (“medium”; Information-seeking) to ≈ 0 (“very small”; Physical activity).

#### Diversion From the Crisis

Chi-square test indicated that activities were different in their efficiency in offering diversion from the crisis [$X_{\left(9\right)}^{2}$ = 9279.27, *p* < 0.001]. The *R*^2^ difference between the unconditional model to the model with the activities effect is 0.19 (“large”). As can be seen in Supplementary Table 3 (model-1) and Supplementary Figure 2, Music was more efficient compared to all other activities except for Entertainment (*p* = 0.11) in obtaining Diversion [all other *p's* < 0.001, *p* (*Hobbies*) = 0.02, Cohen's *d*'s ranged from 0.03 (“very small”; Entertainment) to 1.55 (“large”; Information-seeking)]. Culture by itself was also a significant predictor of activities' efficiency in obtaining the goal of diversion from the crisis [$X_{\left(1\right)}^{2}$ = 4.39, *p* = 0.036, marginal *R*^2^ < 0.01 (“very small”)]. Again efficiency was rated as higher for most activities in collectivist cultures with a few exceptions.

The Culture × Activity interaction term was also a significant predictor of activities' efficiency [$X_{\left(9\right)}^{2}$ = 407.93, *p* < 0.001], but the marginal *R*^2^ difference between models with (Model-3) and without (Model-2) the interaction term is only 0.01 (“small”).

Cohen's *d*'s for differences in activities' efficiencies between cultures ranged from −0.58 (“medium”; Information-seeking) to 0.06 (“very small”; Physical activity).

#### Connecting With Myself and Detachment From the Surroundings

Chi-square test indicated that activities were different in their efficiency to obtain Self-Connection [$X_{\left(9\right)}^{2}$ = 7323.19, *p* < 0.001]. The *R*^2^ difference between the unconditional model to the model with the activities effect is 0.14 (“large”). As can be seen in Supplementary Table 4 (model-1) and Supplementary Figure 3, Music was more efficient compared to all other activities in obtaining Self-Connection [all *p*'s < 0.001, Cohen's *d*'s ranged from 0.10 (“very small”; Hobbies) to 1.49 (“large”; Information-seeking)].

The Culture × Activity interaction term was also a significant predictor of activities' efficiency [$X_{\left(4\right)}^{2}$ = 300.41, *p* < 0.001], but the marginal *R*^2^ difference between models with (Model-3) and without (Model-2) the interaction term is only 0.01 (“small”).

Cohen's *d*'s for differences in activities' efficiencies between cultures ranged from −0.48 (“small”; Information-seeking) to 0.07 (“very small”; Physical activity). The pattern of results was highly similar to the other goals.

To summarize: Music was found to be more efficient than all other activities for obtaining 3 out of the 5 wellbeing goals across cultures: Enjoyment, Venting negative emotions and Self-Connection. Music was also found to be as efficient as Entertainment, and more than all other activities in obtaining Diversion, and as efficient as Hobbies but less efficient than Socializing in obtaining Togetherness. Overall, the activities closest to (but a bit behind) music in terms of efficiency were Entertainment and Hobbies. In general, ratings by collectivistic cultures are higher for most activities as compared to individualistic cultures (especially in Information seeking and Spirituality), but the relative contribution of the activity to obtaining each goal is similar across cultures.

### Did Balcony Singing Influence Feelings of Togetherness?

Supplementary Table 1 (model-1) shows the number of participants that participated in balcony singing per country. Participation varied significantly ranging from 36.1% in Spain (close to it are Italy with 32.2% and Brazil with 26.6%) to 3.8% in Mexico (close to it are Norway with 7.9% and Argentina with 8.0) with the Netherlands (12.3%), Colombia (13.3%), USA (16.4%), United Kingdom (17.0%), and China (18.7%) in between.

To examine if participation in balcony singing affected feelings of togetherness beyond that which may have been engendered by the crisis itself, we created a variable named Emerged-Togetherness, computed as the average of the six items focused on feeling of togetherness during balcony singing, and the average of the five items that asked about “feeling of togetherness during the COVID-19 crisis” (see also Methods section). “Irrelevant” responses were coded as missing values.

We then applied a multilevel regression with Country as a random factor, Participation in balcony as a fixed factor, and Feeling of togetherness as the dependent variable. As seen in Table 5, those participating in balcony singing, felt stronger feelings of togetherness than those who did not (*p* < 0.001 with a medium effect size of 0.06).

**Table 5:** Feeling of “Togetherness” as a function of participation in balcony singing.

**Predictors**	**Estimates**	**std. error**	***p***
**Intercept**	3.13^***^	0.07	** <0.001**
**Participation dummy**	0.54^***^	0.03	**<0.001**
**Random effects**			
σ^2^	0.62		
τ_00__country_	0.05		
ICC _country_	0.07		
N _country_	11		
Observations	4,854		
Marginal *R*^2^/Conditional *R*^2^	0.061/0.125		

*Participation dummy = 1 for participating, 0 not participating*.

*** *p < 0.001*.

### Which Variables Affected Music's Efficiency?

To examine which variables predict the rated efficiency of music, we applied multilevel regressions with respondents nested within countries for each goal. Model-0 in each Table is an unconditional model. Model-1 includes demographic variables (age—with age 18–24 serving as reference, gender, and culture). In Model-2, we added the importance of the goal. In Model-3, the personality variables were added—namely the trait “openness to experience.” Although other personality traits—especially agreeableness—were significantly (though weakly) correlated with some of the goals, the only personality trait to show robust and consistent correlations across all goals was openness to experience. Model-4 added type of music in terms of nostalgia and valence, and finally, in Model-5, we added music's importance in one's life. All the continuous variables were standardized, whilst the categorical variables were dummy coded.

Here we describe in detail results for one goal—Venting negative emotions (Table 6)—and summarize the results for the remaining four goals, details of which can be found in the Supplementary Materials [for Diversion (Supplementary Table 5), Enjoyment (Supplementary Table 6), Togetherness (Supplementary Table 7), and Self-Connection (Supplementary Table 8)].

**Table 6:** Overview of main statistical outcomes of multilevel regression models testing which variables predict the rated efficiency of music in obtaining “Venting negative emotions.”

	**Model-0**		**Model-1**		**Model-2**		**Model-3**		**Model-4**		**Model-5**	
	**Estimates**	**std. error**	**Estimates**	**std. error**	**Estimates**	**std. error**	**Estimates**	**std. error**	**Estimates**	**std. error**	**Estimates**	**std. error**
**Intercept**	−0.03	0.06	0.08	0.10	−0.02	0.09	−0.01	0.09	−0.02	0.08	−0.08	0.06
**Age**												
25–44			−0.08^*^	0.04	−0.08^*^	0.04	−0.09^*^	0.04	−0.08^*^	0.04	−0.04	0.03
45–64			−0.09^*^	0.05	−0.06	0.05	−0.08	0.05	−0.07	0.05	−0.01	0.04
More than 64			−0.09	0.08	−0.05	0.08	−0.06	0.08	−0.08	0.08	0.05	0.07
**Female dummy**			0.04	0.03	0.02	0.03	0.02	0.03	0.03	0.03	0.06^*^	0.03
**Culture dummy**			−0.15	0.13	−0.12	0.11	−0.12	0.11	−0.10	0.10	−0.09	0.07
**Goal importance**					0.21^***^	0.03	0.20^***^	0.03	0.18^***^	0.03	0.11^***^	0.02
**Openness to experience**							0.09^***^	0.02	0.09^***^	0.02	0.02	0.01
**Valence of music**									−0.11^***^	0.02	−0.10^***^	0.01
**Music-induced nostalgia**									0.13^***^	0.02	0.04^**^	0.02
**Music's importance**											0.47^***^	0.02
**Random effects**												
σ^2^	0.97		0.96		0.95		0.94		0.92		0.75	
τ_00__country_	0.04		0.04		0.03		0.03		0.02		0.01	
ICC _country_	0.04											
N _country_	11		11		11		11		11		11	
Observations	3,922		3,922		3,922		3,922		3,922		3,922	
Marginal *R*^2^/Conditional *R*^2^	0.000/0.041		0.008/0.051		0.025/0.056		0.034/0.061		0.057/0.080		0.240/0.252	

*Culture dummy = 1 for individualistic culture, 0 for collectivistic culture. Age reference is 18–24*.

*Female dummy = 1 for female, 0 for males*.

* *p < 0.05*,

** p < 0.01, and

*** *p < 0.001*.

As can be seen in Table 6, the strongest predictor of music efficiency in Venting Negative Emotions, was the importance of music in one's life [β = 0.47, *p* < 0.001; marginal *R*^2^ = 0.18 (“large”)]. Other significant predictors are Goal importance, the personality trait Openness to experience, and the type of music, but their relative contribution to the model diminishes significantly when we add Music importance (compare Models 2–4 to Model 5). Interestingly, while choice of nostalgic music contributes positively (though weakly) to ratings of efficiency of music for Venting negative emotions, music valence contributes negatively indicating that those choosing music defined by them as more pessimistic, rate music as less efficient. Moreover, contrary to other predictors, this variable is not influenced by the addition of the variable of Music importance in one's life. We will discuss variables related to the type of music more below.

Very similar results are shown in Supplementary Tables 5–8 with the predictive power of music importance ranging from β = 0.48 [marginal *R*^2^ = 0.20 (“large”)] for the goal of Self-connection, to β = 0.37 [marginal *R*^2^ = 0.12 (“medium-large”)], for the goal of Togetherness, with a similar pattern of contribution of the other variables. The only goal for which Culture was a significant predictor was the goal of Togetherness (Supplementary Table 7 and Table 7). Collectivistic cultures rated music as more efficient in obtaining this goal as compared to individualistic cultures [β = −0.28, *p* < 0.01; Cohen's *d* = 0.24 (“small”)] and this contribution was only somewhat diminished when adding other variables to the model including the addition of Music importance (Model-5, β = −0.22 *p* < 0.001). Another finding to note is the contribution of age to predicting the efficiency of music, but only for obtaining Enjoyment or Togetherness as described below. Gender did not have an effect on any of the goals and therefore was not included in the following analyses.

**Table 7:** Overview of main statistical outcomes of multilevel regression models testing the effects of Age, Culture, and Age × Culture on rated efficiency of music in “Reducing loneliness and creating a sense of togetherness.”

**Predictors**	**Model-0**		**Model-1**		**Model-2**		**Model-3**	
	**Estimates**	**std. error**	**Estimates**	**std. error**	**Estimates**	**std. error**	**Estimates**	**std. error**
**Intercept**	5.71^***^	0.08	5.61^***^	0.09	5.80^***^	0.10	5.79^***^	0.10
**Age**								
25–44			0.07	0.05	0.07	0.05	0.09	0.07
45–64			0.20^***^	0.06	0.20^***^	0.06	0.21^**^	0.08
More than 64			0.42^***^	0.09	0.42^***^	0.09	0.31^*^	0.15
**Culture dummy**					−0.35^**^	0.13	−0.33^*^	0.14
**Age × Culture**								
25–44 **×** Culture							−0.06	0.10
45–64 **×** Culture							−0.01	0.11
65+ **×** Culture							0.16	0.19
**Random effects**								
σ^2^	1.48		1.47		1.47		1.48	
τ_00__country_	0.07		0.07		0.04		0.04	
ICC _country_	0.05		0.04		0.03		0.03	
N _country_	11		11		11		11	
Observations	4,012		4,012		4,012		4,012	
Marginal *R*^2^/Conditional *R*^2^	0.000/0.045		0.007/0.051		0.024/0.050		0.025/0.050	

*Culture dummy = 1 for individualistic culture, 0 for collectivistic culture. Age reference is 18–24*.

*Female dummy = 1 for female, 0 for males*.

* *p < 0.05*,

** p < 0.01, and

*** *p < 0.00*.

### Efficiency of Music by Age and Culture

Since age emerged in the previous analysis as a relevant predictor of music's efficiency in the goal of Togetherness and Enjoyment, we further analyzed this effect by examining its interaction with culture. We applied multilevel regression with respondents nested within countries for each goal. Model-0 is the unconditional model, Model-1 includes the main effect of Age, Model-2 includes also the effect of Culture, and Model-3 includes the Age × Culture interaction. In the following models, all the predictors are categorical and were dummy coded. The reference age-group is 18–24.

Table 7 shows the results for the goal of “Reducing Loneliness and Creating a Sense of Togetherness” (the relevant table for the goal of Enjoyment is presented in the Supplementary Table 9).

**Figure 4:**
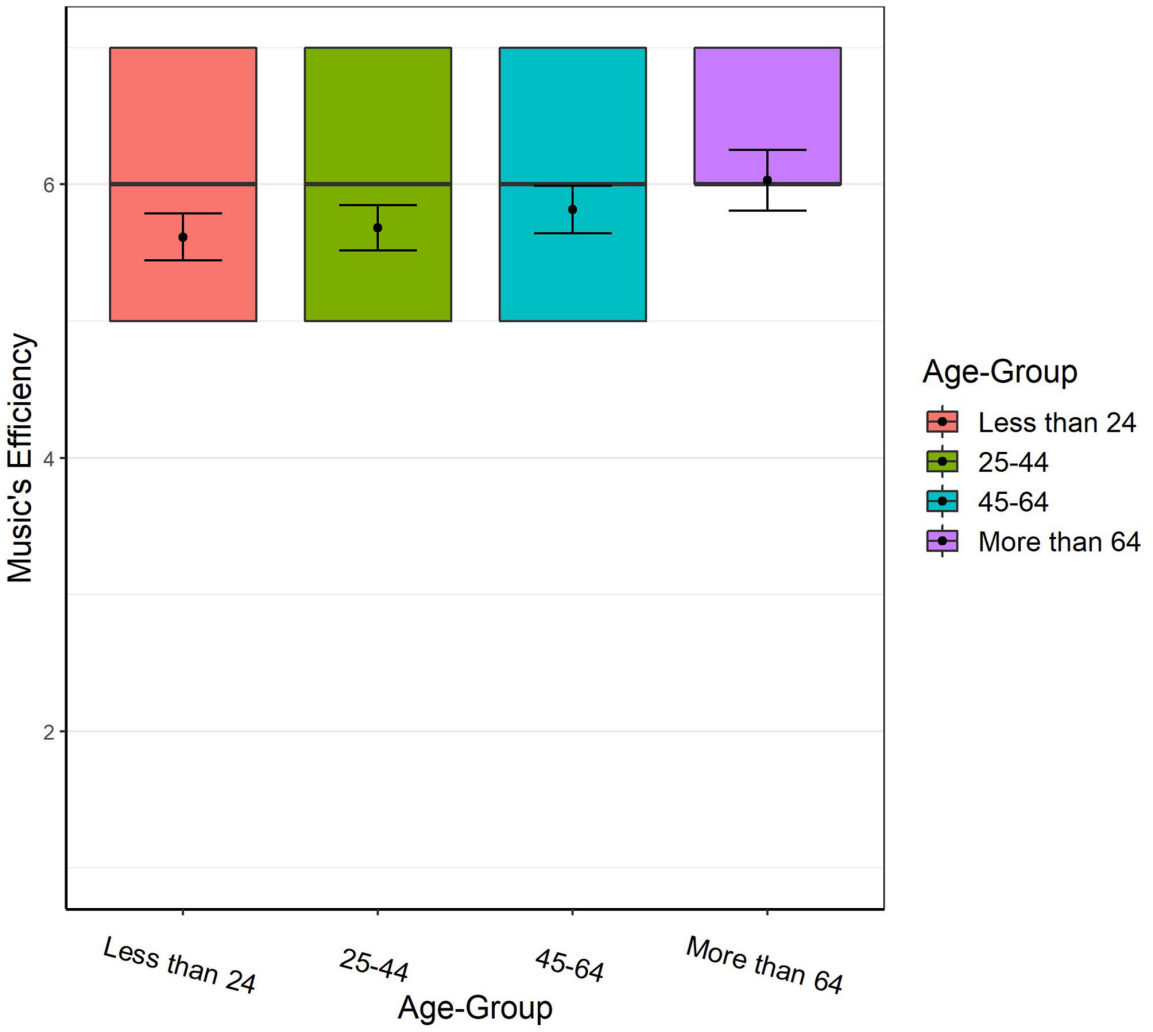
Efficiency of music in obtaining Togetherness by age. Error bars represent 95% CI. Colored squares represent the 2nd and 3rd quartiles of responses. Black lines represent the median, and dots represent the mean.

For Togetherness both Age [$X_{\left(3\right)}^{2}$ = 27.87, *p* < 0.001] and Culture [$X_{\left(1\right)}^{2}$ = 6.61, *p* = 0.010] were significant predictors of music's efficiency. The effect-sizes were 0.01 (“small”) for age and 0.02 (“small”) for culture (see Table 7 and Figure 4). *Post-hoc* pairwise comparisons with Bonferroni correction revealed that music was significantly more efficient for the age group of Over 64 compared to the age-groups of Under 24 [Cohen's *d* = 0.34 (“small”)] and 25–44 [Cohen's *d* = 0.28 (“small”)], and for the age-group of 45–64 compared to the age-groups of Under 24 [Cohen's *d* = 0.16 (“very small”)] and 25–44 [Cohen's *d* = 0.10 (“very small”)] in obtaining Togetherness. There was no interaction between the variables of Age and Culture.

For Enjoyment (Supplementary Table 9), Age was the only significant predictor of music's efficiency [$X_{\left(3\right)}^{2}$ = 15.40, *p* = 0.002] with effect-size <0.01 (“very small”; see Supplementary Table 9 and Supplementary Figure 4). *Post-hoc* pairwise comparison with Bonferroni correction, revealed that music was significantly more efficient for the age group of 18–24 compared to the age-groups of 25–44 [Cohen's *d* = 0.11 (“very small”)] and 45–64 [Cohen's *d* = 0.14 (“very small”)] in obtaining Enjoyment.

### Influence of Distress on Obtaining Wellbeing Goals Through Music

Our results indicate that music is highly efficient for obtaining most of the wellbeing goals defined in the current study regardless of gender, and only marginally influenced by age and culture. However, while this may be true for the general population, one can ask whether the same results would be obtained in individuals experiencing higher levels of worry, stress, anxiety, and depression. To examine the association between distress and wellbeing goals, we averaged scores from the three subcomponents of the DASS questionnaire (depression, anxiety, and stress), and the six items related to worry about COVID−19 (“how much do you worry about dying, getting infected, infecting others” etc. —see Methods). We first examined which goals were more or less related to distress levels by calculating the correlation between this variable and ratings of goal importance. Pearson correlation coefficients ranged from *r* = 0.17 and 0.14 (*p* < 0.001) for Venting and Diversion, respectively, to −0.03 (*p* > 0.5) for Enjoyment, indicating that diversion and release of negative emotions are more important for those higher in distress.

We then examined whether people higher in distress chose specific types of music in terms of arousal (more relaxing?), valence (more negative?) or nostalgia (more nostalgic?) and whether they felt music was useful in obtaining the goals of Venting negative emotions and Diversion. Distress was correlated with emotional valence of music (higher distress was associated with more “pessimistic music” *r* = 0.17, *p* < 0.001) and with music inducing nostalgia (*r* = 25 *p* < 0.001), but less so with the dimension of arousal (*r* = 0.07, *p* < 0.001). In contrast, there was no correlation between Distress and ratings of music efficiency in obtaining the goals more important for this group (*r* = 0.01 for Venting and *r* = −0.04 for Diversion). That is, those experiencing higher levels of distress did not find music to be especially efficient or inefficient in releasing their negative emotions.

**Figure 5:**
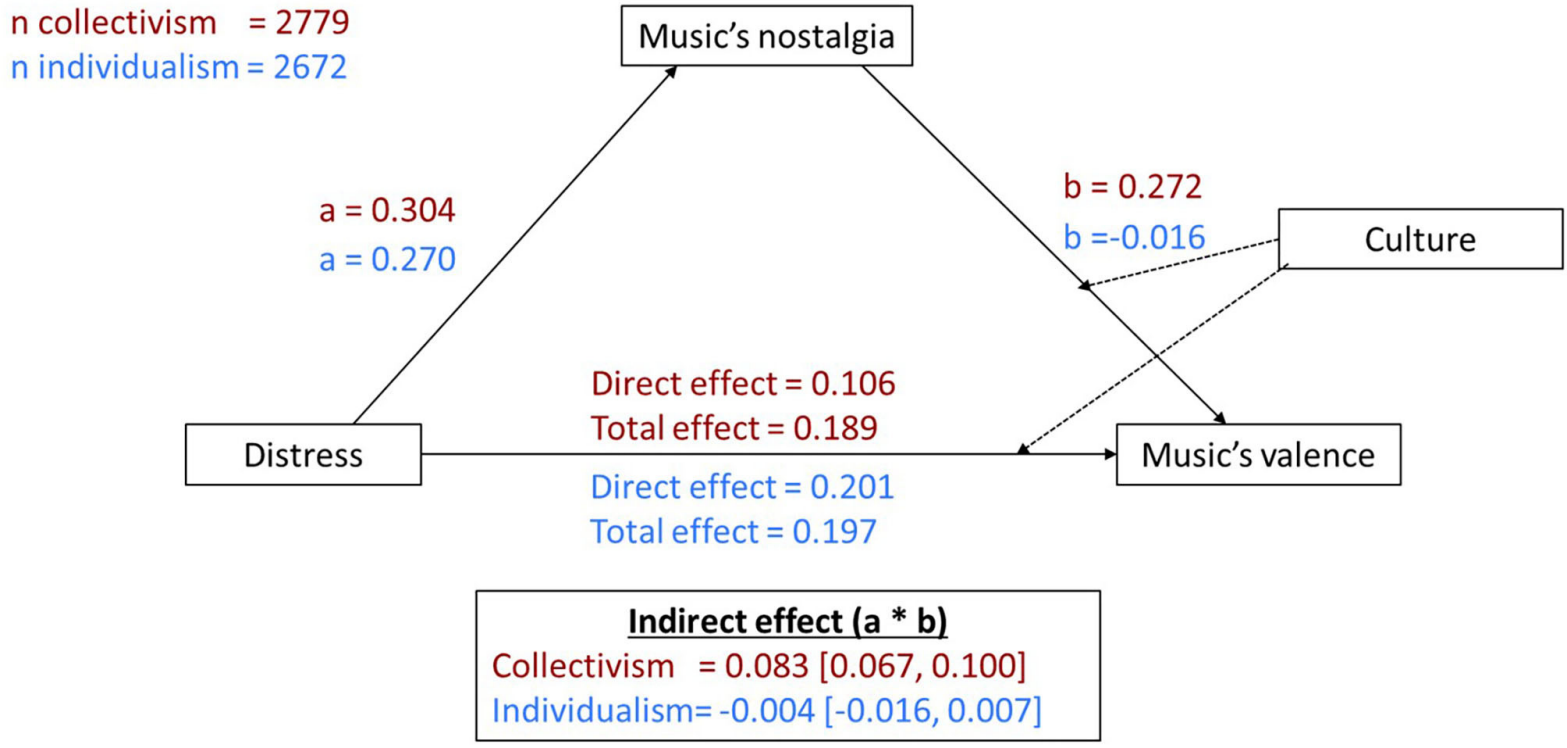
Moderated-mediation model. X, distress; Y, music valence; Mediator, music nostalgia; Moderator, culture.

In order to further examine the relationship between Distress and choice of music in terms of Music Valence and Music Nostalgia, we tested a mediation model based on Preacher and Hayes' (2004) bootstrapping method with Nostalgic music as mediator of the effect of Distress on music's Valence and Culture as a moderator (see Figure 5). In the model, we assumed that Culture may moderate each one of the paths in the model (*a, b, c*′), and any combination of them, where c is the total effect of Distress on Music Valence, and c′ represents the direct effect without mediation. All variables were standardized before the analysis.

For *path-a* (relationship between Distress and Music's Nostalgia), the difference between Cultures was insignificant (0.03, Bootstrap CI = [−0.02, 0.09]). In contrast, for *path-b* (0.29, Bootstrap CI = [0.23, 0.35]) and *path-c*′ the differences between Cultures were both significant (−0.10, Bootstrap CI = [−0.16, −0.03]), indicating a stronger relationship between music's nostalgia and negative music valence for collectivist cultures, Indeed, the coefficient of *path-b* was moderated by a significant indirect effect in collectivistic cultures (a*b = 0.083, Bootstrap CI = [0.07, 0.10], a*b/c = 0.44), but not in individualistic cultures (a*b ≈ 0.00, Bootstrap CI = [−0.02, 0.01], a*b/c ≈ 0.00). That is, while in collectivistic cultures some of the effect of Distress on choosing more negatively valenced music is explained via their choice of nostalgic music, in individualistic cultures there is no such mediation.

## Discussion

The current study asked whether music is an efficient activity for achieving goals related to wellbeing under extreme stress conditions, across cultures, ages, and genders. We also asked whether music would fare better than other daily activities that are as easily accessible as music in coping with the situation. We found positive answers to both questions. Moreover, this seemed to be widespread across goals and universal among cultures: music was more efficient than all other activities at achieving most goals. Effects of age and culture were small, and no gender differences were observed.

### Wellbeing Goals Are Relevant and Similar Across Cultures

First, our analyses validated the wellbeing goals we defined as relevant for coping with the lockdown. Enjoyment was rated as the most important goal, followed by venting negative emotions, self-connection, togetherness, and finally diversion. Interestingly, self-connection was one of two goals in which culture played a role: participants from collectivistic cultures rated this goal significantly higher than individualistic cultures. This may be due to the double pressure on defining a physical and psychological space within the group during the lockdown. Diversion was the only goal which was rated higher by participants in individualistic as opposed to collectivistic cultures. This may be related to cultural differences in the need for information about the crisis, as participants from collectivistic cultures indicated that they value information seeking relatively more to address most wellbeing goals.

### Music Is the Most Efficient Activity in Obtaining Diverse Wellbeing Goals Across Age, Gender, and Culture

Our study confirms findings reported in the literature that music is highly efficient in regulating mood, defining self-identity, followed by reducing loneliness and creating a sense of togetherness.

Music was found to be most efficient at attaining the goal of enjoyment and maintaining a good mood. This goal was also most often cited by participants in studies of music-use as the most important reason for listening to music (Juslin and Laukka, 2004; Zentner et al., 2008, Schäfer and Sedlmeier, 2009; Ter Bogt et al., 2011; Lonsdale, 2019). However, note that what we measure is the perceived efficiency of music to obtain this goal—therefore drawing one step closer to the connection between music and the functions it is assumed to fulfill. Recently, Saarikallio et al. (2019) identified factors underlying positive emotions related to music, the strongest of which were relaxation, power, joy and to a lesser degree kinship—all seem especially relevant during the COVID-19 crisis. Interestingly, and contrary to some studies, the youngest age group in our sample (18–24) rated music as most efficient for enjoyment. Previous studies noted that young adults often use background music for daily tasks (Bersch-Burauel, 2006), and to enhance both positive and negative emotional experiences (North et al., 2000). In contrast, older adults often use focused listening, with a preference for positive emotions likely due to their links to autobiographical memory (Mather and Carstensen, 2005; Laukka, 2007).

Our findings may reflect specific challenges imposed by lockdown and social distancing, with loneliness being a central concern for older adults (over and above mood regulation). Indeed, although music was not the most efficient activity for obtaining the goal of togetherness (compared to socializing), older adults rated music as more efficient in obtaining togetherness, suggesting they were using music relatively more for this goal. While music has often been cited as an especially useful medium for social bonding and group cohesion (Savage et al., 2020), this has usually been associated with group musical activity such as singing, playing, or drumming (Tarr et al., 2014; Pearce et al., 2015), or with group-listening activities such as going to a concert together (Papinczak et al., 2015). As these group activities were all restricted during lockdown, or were challenging to conduct due to present technology, the present findings pertain directly to the use of music as a possible surrogate for the positive effects of social stimulation on wellbeing.

In times of social isolation, people may use different strategies to relieve loneliness, such as creating parasocial relationships with media (e.g., TV series) characters, or adopting a perspective of a character in a book thus creating a sense of belonging. Schäfer and Eerola (2020) showed how this coping strategy, called the “surrogacy hypothesis,” could also apply to music. They found that listening to music (even in isolation) can offer a sense of comfort and belonging, a sense of shared feelings and recall of significant others, thus reducing loneliness (see also Groarke et al., 2016). Indeed, music can be experienced as a virtual person through its ability to arouse and express emotions and movement. These qualities can be experienced by us automatically (through contagion, the mirror neuron system, and similarity with emotional prosody), or through an imaginative act (e.g., the “persona theory”) inducing a feeling of a human presence (Watt and Ash, 1998; Cochrane, 2010). This experience may be even stronger if the music is vocal. In our study we found that even though music was, as expected, less efficient than social interactions (real or virtual) in creating a sense of togetherness and reducing loneliness, participants rated it as more efficient than all other activities, except for hobbies (or “things I like to do”), which were as efficient as music. This includes activities that potentially could be performed together and thus enhance a sense of togetherness including food (eating together), doing productive things (e.g., cleaning together), or physical activity.

Since the pandemic began, balcony singing has received high visibility in the media (Grahn et al., 2020) as a unique activity that can create a sense of togetherness. Our findings clearly indicate that this activity enhanced the mood of its participants and their feeling of togetherness; thus, it seems to be an excellent mood-regulating and social-bonding activity. Nonetheless, we found it was significantly used (~30%) only in a small number of countries (Italy, Spain, and Brazil), with all other countries using it to a much lesser extent.

While lockdown rules imposed situations of great loneliness for some people, for others it created a lack of privacy, and threat to self-identity due to loss of social connections, work, and other activities which define who we are. Here we found that music was more efficient than all other activities in obtaining the goal of “connecting to myself and detaching from my surroundings.” This converges with prior work showing that music can serve as a medium for defining and expressing our own particular identity through our choices of music and its presence in our autobiographical memories (DeNora, 1999; Laiho, 2004; Janata et al., 2007). We also found that music was the most efficient activity to relieve negative emotions. While stress and uncertainty are common sources of negative emotions associated with the pandemic, music has well-replicated effects on reducing stress, both as indexed by its psychophysiological correlates (Fancourt et al., 2014) and psychological experience (e.g., anxiety, nervousness; de Witte et al., 2020) across multiple contexts (Bulfone et al., 2009; Kushnir et al., 2012; Linnemann et al., 2015; Kappert et al., 2019).

In addition to music, entertainment, and other hobbies were the best activities for diversion from the crisis. These activities were closest to music in obtaining most goals, suggesting common functions such as enjoyment, emotional or intellectual engagement, and negotiating self-identity (Rentfrow and Gosling, 2003, 2006; Lonsdale and North, 2011). Yet, considering the wide varieties of richer visual and semantic content in most entertainment media and the wide definition of the category “things I like to do” (e.g., hobbies, pets), it is remarkable that music is on par with these other activities for diversion and better at obtaining most other goals.

Ratings of music's ability to obtain all these goals were no different across genders. This is consistent with studies that found only negligible differences in music use as a function of gender (Schäfer and Sedlmeier, 2009; Ter Bogt et al., 2011), although other studies did find some such differences (Lonsdale and North, 2011; Kuntsche et al., 2016; Greb et al., 2018; Gupta, 2018; Lonsdale, 2019). These discrepant results may stem from the type of function under consideration: while the studies that found gender differences focused on arousal, dance, social bonds, expression of emotions at various points along the lifespan, the current study focused on more abstract and general goals as they related to wellbeing during the lockdown.

### Cultural Differences in Uses of Music

We also found few differences between collectivistic and individualistic cultures in music use for obtaining the various goals. Music was evaluated consistently high in efficiency, and the rank order of activities for specific goals was consistent across cultures. The main difference we did find—higher ratings of music efficiency in collectivistic compared to individualistic cultures for obtaining a feeling of togetherness—was not specific to music and could be seen across all activities. Indeed, in general, a main effect of culture was found, with higher ratings of effectiveness of activities in collectivist cultures, and only very small interaction effects. This may be because togetherness is more valued in collectivistic cultures (Oyserman et al., 2002). Previous cross-cultural studies have shown that higher collectivism is related to lower loneliness and higher togetherness across cultures (Heu et al., 2019), and as a result, there is a greater need to reduce feelings of loneliness by engaging in various activities, including music, for those from collectivistic cultures. This is in contrast with Juslin et al. (2016) who showed that individualistic cultures (in 3 countries) rated their use of music for most music-related functions including “relax,” “emotion,” and “pastime” higher than collectivistic cultures. These differences were not evident under the challenging conditions of the COVID-19 pandemic. Our findings show a universal use of music to obtain wellbeing goals during times of crisis, and by that, extend previous findings for universal functions of music across a large number of traditional communities (Mehr et al., 2019). Indeed, Juslin et al. state that although they did find differences between individualistic and collectivistic cultures, these differences were small, such that cross-cultural similarities were larger than cultural differences (see also Saarikallio et al., 2020). Our findings largely echo this claim.

### Importance of Music Predicts Ratings of Music

Ratings of music's importance in one's life strongly predicted ratings of music efficiency. It is difficult to draw an arrow of cause-and-effect: is music important because it helps achieve wellbeing goals? Or does music help achieve wellbeing goals because of its perceived importance? Despite the obvious connection between the two, only a few studies showed a direct connection between beliefs about music and its ability to have a physiological or psychological influence on us (Kreutz et al., 2008). Our result converges with a prior study which showed that the more participants value music in their lives, the higher they rated their use of music for mood enhancement, coping, and self-identity (Ter Bogt et al., 2017). This study posited that listeners “can be regarded as people who have discovered how to use music to help face the developmental tasks and difficulties, that, to a certain extent, appear in everybody's lives” (p. 158). A related study found a correlation between importance of music in one's life and ratings of functions of music, concluding that the greater the benefit people assigned to music, the better they liked it, thus hinting at a positive feedback loop between the importance of music and its use for wellbeing (Schäfer and Sedlmeier, 2009).

Our study also shows that importance of music in one's life partially mediates other variables which predict the ratings of music efficiency, as could be deduced from the reduction in their predictive power when music importance was added to the model. One such variable is the personality trait of Openness to experience, which has been linked with an increased sensitivity to experience pleasurable chills when listening to music (Nusbaum and Silvia, 2011), a tendency to listen to more diverse and complex styles of music (such as classical and jazz; Rentfrow and Gosling, 2003), as well as liking music expressing negative emotions such as sadness and fear (Vuoskoski and Eerola, 2011).

### Distress and Culture Are Associated With Music Valence and Nostalgia

Across the entire sample, listening to more pessimistic music was associated with lower ratings of music efficiency. This pattern, in contrast to other variables, was not influenced by the importance of music in participants' life. We intentionally avoided use of the term “sad” music in our questionnaires since this is often associated with positive feelings of awe, empathy, beauty, and feeling moved (Vuoskoski and Eerola, 2012; Sachs et al., 2015). Therefore, a possible interpretation is that listening to negatively valenced music elicited negative emotions of pessimism or despair, thus perpetuating the negative mood (Saarikallio and Erkkilä, 2007; Garrido and Schubert, 2013). Indeed, listening to more pessimistic music was associated with distress, which in turn was associated with nostalgia.

Nostalgia, with its bitter-sweet qualia, is often associated with autobiographical memory, high arousal, familiarity, liking, and strong mixed emotions (Michels-Ratliff and Ennis, 2016), especially in sad music (Vuoskoski and Eerola, 2012). Individual differences in personality and affective states influence the experience of nostalgia through music (Barrett et al., 2010), as well as elements in the lyrics of the song (Batcho, 2007). However, little is known about the effects of nostalgic music on wellbeing. Studies show that nostalgia can be triggered by negative life events, or by awareness of a limited time horizon. The COVID-19 pandemic has unsurprisingly brought about a rise in consumption of nostalgic cultural and media artifacts such as films, TV series, sports matches from the past, games, and music (Gammon and Ramshaw, 2020; Grahn et al., 2020; Lee and Kao, 2020; Martin, 2020; Yeung, 2020). This trend can be explained not only as a means for coping with feelings of threat, discontinuity, and restrictions on freedom, but also as a result of lockdowns with families spending more leisure time together than ever before (Gammon and Ramshaw, 2020). A recent analysis of Spotify data across six European countries (Yeung, 2020) showed an absolute rise in the consumption of nostalgic music. Our data extend these findings by showing a connection between distress and negative music, which is mediated by nostalgia. Interestingly this mediation is only found in collectivistic cultures but not in the individualistic ones. This is consistent with one of the largest-scale cross-cultural studies on music use (Juslin et al., 2016). The authors showed that individuals from collectivistic cultures report experiencing nostalgia more frequently and rate music arousing the experience of nostalgia as more important in comparison to individualistic cultures. Moreover, and consistent with our findings, negative memories were more associated with nostalgia in collectivistic cultures, suggesting that “unhappy memories can also evoke nostalgia, if the reminiscence heightens one's sense of self and connectedness to other people” (p. 307). We suggest that the link between negative memories and nostalgia could be enhanced in times of distress and imposed social distancing, particularly if nostalgic memories are of collectivistic nature such as music played in social contexts. Another interpretation could stem from subtle differences in the usage of the word “nostalgia” in different languages—even when the same word itself is used. For example, Farese and Asano-Cavanagh (2019), exemplify using discourse analysis how the English and Italian usage of the word *nostalgia* differs in intensity and color, with the negative feeling of Nostalgia possibly more pronounced and more extreme in Italian. Dissociation of these two possibly interrelated interpretations awaits further study.

## Limitations and Future Directions

As participants were recruited through convenience sampling and snowballing, they cannot be considered as fully representative of a population, and more work is needed to reach populations who remain underrepresented such as people of older age, people with disabilities, people from ethnic minority groups, and people who do not have access to the internet. The findings of subtle variations found with age, culture, and levels of distress, indicate the relevance of taking into account individual differences when considering the effects of music on wellbeing. Moreover, since the invitation to participate in the study mentioned we were interested in the effect of daily activities “especially music” on wellbeing goals, our sample may have been biased toward those with a special affinity for music. Nonetheless, our results are totally consistent with a recent study showing the superiority of music listening in obtaining wellbeing goals over other activities, during the COVID-19 pandemic, in a different sample not especially inclined toward music (Mas-Herrero et al., 2020).

An interesting yet unresolved question is the relative contribution of different music activities to wellbeing in the current crisis. Naturally, we focused on music listening—the most widespread and hence possibly also the most relevant (~46% of our respondents had no training at all and another ~24% <6 years—hence at most amateurs). Yet, in the questionnaire we defined the music activity category as wide as possible indicating this activity could also include singing and playing. While it could have been useful to further breakdown the type of music activity to its subcomponents we did not do so, in order to keep the questionnaire short enough (~15 min) to ensure high response rate.

In order to really be able to understand the relative contribution of different musical activities to the specific challenges presented by the pandemic, lockdown, and social distancing, there is a need for a separate in depth study (on-line rather than retrospective), targeting only the activity of music and looking separately at three different groups: Listeners, participants in some music activity in an amateur fashion, and music professionals. Each of these may use music somewhat differently and for different purposes in regular times, and possibly even more diversely during times of crises. As listeners, each time we select a given piece or playlist, we consciously or unconsciously evaluate our mood and act upon it (MacDonald, 2013), hence engage in some “self-therapy” which in times of stress and anxiety could be extremely important (as in fact our study shows). But even for this group we do not have comprehensive data about the context of listening beyond the important fact that participants largely listened to self-selected music. Did they listen alone or with others in their home? What was their degree of engagement with the music? Did they listen to music as a sole activity? Or as background to other activities, and if so which? Did they sing along? Moved to it? Did they listen over headphones? This and other contextual data could be relevant for the functions fulfilled by music (Greb et al., 2018). As for amateurs, assuming many community and music group activities could not take place physically during the lockdowns, it could be interesting to study how this affected those participating regularly in such activities, and which types of substitutions did they find if any (see below). For those practicing music at home, one can assume they would enjoy the wellbeing benefits of music reported by listeners (but possibly more intensely due to a more intense and physical engagement with the music), with additional benefits such as obtaining a sense of achievement, self-esteem and fulfillment (Krause et al., 2018).

Interestingly ~31% of our participants (Supplementary Table 1) attempted to use some electronic platform for joint music activities, and somewhat unexpectedly, 50% of these reported the experience as “different but with its own benefits,” suggesting that distant music making could potentially become a relevant new medium. This resonates with our results showing improved mood and increase in feelings of togetherness (akin to solidarity) following balcony singing. Both findings, could have implications for use of music as a useful medium for social bonding under the imposed social distancing, but require a more in-depth analysis which could ultimately potentially inform policy makers. Clearly, more work is needed to understand the special benefit of music making as compared to music listening in the current circumstances.

Nonetheless, even music listening was clearly shown to be more efficient than most other comparable leisure activities at achieving goals for wellbeing during the pandemic. This has policy implications, for example with regard to the allocation of financial resources: While policymakers worldwide turn to stimulus packages to combat the economic effects of the pandemic (e.g., https://www.statista.com/statistics/1107572/covid-19-value-g20-stimulus-packages-share-gdp/), it is important to note that allocating financial resources toward public musical activities may help societal wellbeing, over and above supporting the welfare of music makers.

The survey further offered rich insight into relationships between activities, wellbeing, and culture. Despite general similarities across countries in the challenges imposed by the COVID-19 pandemic, there are many differences in severity of the pandemic and of measures taken to cope with it. Importantly, the survey was gathered in the months of July to– October 2020 with some countries experiencing relief after the first lockdown, others still in lockdown, and yet others seeing a rise in cases toward a second lockdown. Therefore, results for specific countries may show patterns that deviate from the overall results presented here (see Martínez-Castilla et al., 2021).

Our comparison between collectivistic and individualistic cultures showed some promising differences that warrant further research and a more refined selection of countries. Differences between collectivistic and individualistic cultures were strongest in the perceived effectiveness of information seeking and spirituality. The latter difference may also be relevant for the function that music may serve in times of crisis and is in line with previous findings of the larger role of spiritual experiences in response to music in collectivist cultures (Juslin et al., 2016). Otherwise, the effectiveness of music and other activities was rated very highly in both types of cultures, and often overall even higher for collectivist cultures than for individualist cultures. This is an important confirmation of the perceived relevance of music across cultures. It should be noted that this perceived relevance did correlate with the importance of music in participants' lives more generally, which tended to be high for a great proportion of our participants. In our view, of special interest for future studies, is the relationship between distress, wellbeing goals, and the type of music listened to. One possibility is that cultures may differ in their use of music to evoke nostalgia, emphasizing negative valence in a manner that may be adaptive vs. maladaptive, depending on the context. Analyses on the effects of culture may also benefit from moving beyond the collectivism vs. individualism constructs, for example, by accounting for variation in lockdown measures, COVID-19 related morbidity, cultural uses of music, demographic characteristics, and the living circumstances of participants.

### Conclusions

The current report is the most comprehensive cross-cultural examination to date on the uses of music during COVID-19 and its ensuing lockdown. We investigated the use and perceived effectiveness of music to address wellbeing goals. Our survey provided an efficient means to reach a large number of participants across countries in a short period of time. Results showed a prominent use of music to achieve all assessed goals of wellbeing and highlight music as an effective means to facilitate social connectivity and regulate mood and emotion across cultures. We hope this and other studies will inform policy makers about using music as an accessible, low-cost, and highly enjoyable activity in the upcoming months in which mental-health and solidarity will probably be a major challenge.

## Data Availability Statement

The raw data supporting the conclusions of this article will be made available by the authors, without undue reservation.

## Ethics Statement

The studies involving human participants were reviewed and approved by the ethics committee of the Humanities at the Hebrew University of Jerusalem Israel. The participants provided their written informed consent to participate in this study.

## Author Contributions

RG and DS provided initial conception, organization, and main writing of the text. BC and SI analyzed the data and prepared all figures and tables. All other authors were involved in data collection for their respective countries and acted as consultants and contributors to research design, data analysis, and text writing. They read and approved the draft.

## Conflict of Interest

The authors declare that the research was conducted in the absence of any commercial or financial relationships that could be construed as a potential conflict of interest.
