# Supplementary material for: “Help! I Need Somebody”: Music as a Global Resource for Obtaining Wellbeing Goals in Times of Crisis
Source: Front Psychol. 2021 Apr 14;12:648013. doi: 10.3389/fpsyg.2021.648013 (PMC8079817; doi:10.3389/fpsyg.2021.648013)
Supplement: Supplementary file 1 [file Data_Sheet_1.docx]

Appendix 1: The situation in terms of the COVID-19 pandemic and measures taken to cope with it in participating countries during the data collection

**Covid-19 situation in China**

Data collection in China started since the first week of August and ended on 30th Sep. By 30th Sep, the National Health Commission had received reports of 85414 confirmed deaths and 4634 death in mainland China (<http://en.nhc.gov.cn/2020-10/01/c_81792.htm>). The pandemic broke out in the city of Wuhan in Dec 2019, which rapidly spread from Wuhan to the entire country in just 30 days (see the figure below). Meanwhile, Chinese Lunar New Year national holiday was scheduled from 25 Jan onwards, whereas the extended holiday during which attendance at school and work was prohibited. On 8th April, the city Wuhan unsealed, which is the symbolic significance to the overall situation of notional epidemic prevention and control. Since then, the new cases are mainly imported cases and China has entered to the stage of normalization of epidemic prevention and control. The focus of P.R.C Government switched to the psychological counseling for cured patients and the promotion of comprehensive rehabilitation of cured patients. (<http://www.nhc.gov.cn/jkj/s7914/202009/d2a588a1c3c048c4a7a62e06279a17b5.shtml>).


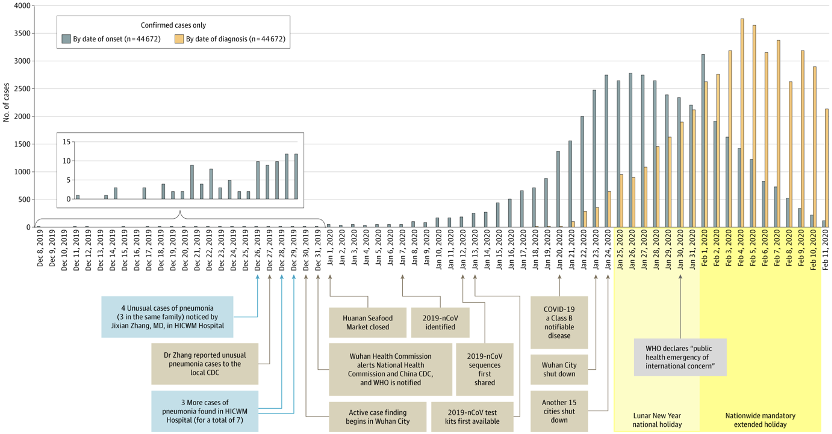


**Figure 2. Epidemic Curve of the Confirmed Cases of Coronavirus Disease 2019 (COVID-19).**  Adapted from Wu & McGoogan (2020)

**COVID-19 situation in USA**

As of October 16, 2020, the USA has recorded 8,228,865 cases and 222,953 deaths from COVID-19. The pandemic peaked in three waves in the USA to date, with the first wave in March, second wave in July, and a third wave as of current writing (October 2020). There are significant regional variations in the severity and rates of transmission across the US. While the first wave focused in the Northeast, the second wave focused on the South and the Midwest, and the third wave is widely in many states. The highest rates of infection are in more populous states including New York, California, Florida, and Texas, whereas less populous states such as Vermont and Maine have relatively few cases. Lockdown rules include travel restrictions and the closing of schools, businesses, and public gathering spaces. These lockdown rules are mandated by state governments and thus vary by state.

**COVID-19 situation in UK**

Data was collected in the UK from 28 July until 6 November 2020. At the end of July, the total death count was as high as 41,135, which remained relatively stable until the start of October. From October, the number of deaths started to increase more rapidly from 42,202 to 46,555 at the end of the month. On 6 November, the number of deaths had increased to 48,475 and a new national lockdown was imposed in England. The development in the number of deaths illustrates the development of the pandemic. In July, the country was emerging from a strict lockdown over the spring, where schools had been closed, and social interaction had been severely restricted. This had effectively lowered the number of daily cases. From September and October onwards, the pandemic was increasing in strength leading to new measures to be put in place, which were differentiated between regions with stricter and less stringent restrictions, depending on the rate of infections and the number of daily cases (so-called three tiers system in England). The situations in Scotland, Wales and Northern Ireland were similar to the UK, although with some variation in timing and type of restrictions that were implemented in the Autumn.

**COVID-19 situation in Spain**

As of September 26, 2020, Spain has recorded 12,272 cases and 114 deaths per million from COVID-19. The pandemic peaked in two waves in Spain to date, with the first wave starting in March and ending in June, and a second wave starting in September as of current writing (December 2020). There were significant regional variations in the severity and rates of transmission across Spain during the first wave, but not in the second one, with few exceptions. The first wave affected the most in more populous cities and their surroundings as Madrid, Barcelona and Valencia. Lockdown rules during the first wave, mandated by the national government, were very severe: confinement at home and only essential working places were open. Data gathering for this study was carried during the summer holiday (August) and the return to work and school (September). The prevention measures were social distancing, facial coverings in all public spaces and limitations in public gathering spaces. The rates of infection started to increase in August, and September, originating the second wave.

**COVID-19 situation in Italy**

Data was collected in Italy from July until the end of September 2020. As of September, Italy has recorded 314861 cases and 35894 deaths from COVID-19. The pandemic peaked in two waves in Italy to date, with the first wave starting in February and ending in June, and a second wave starting in September as of current writing (December 2020). At the beginning of March, due to a rapidly growing number of SARS-CoV-2 cases, Italy was the first European country to impose severe restrictions on the population in an attempt to reduce the increasing diffusion of the virus and the mounting pressure on the national health system. Such restrictions initially involved the northern regions, the most severely hit by the virus, but were soon extended to the rest of the country. People were not allowed to move, except for work (only the industries of national significance and health sector), health reasons and purchase of essential goods. Such mitigation measures were enforced in Italy in their strictest version until the beginning of May (“Phase 1” Lockdown). These measures were followed by a “Phase 2” Semi-Lockdown in which restrictions were gradually reduced according to the virus diffusion. The rates of infection started to increase in August, and September, originating the second wave.

**Covid-19 situation in The Netherlands**

In The Netherlands, the government implemented a so-called “intelligent lockdown” on March 15th, initially closing schools, as well as any other place where more than 30 people could convene (including all restaurants and bars, gyms, theaters) but not public transport or any stores. While schools opened again in early May, most other measures were not lifted until July 1st, after which people were only still encouraged to keep distance and wash their hands. In the period up until July first, 50276 people were registered as tested positive, 11065 people were hospitalized and 6150 people died. While infections stayed relatively negligible in July and the first half of August, from mid-August through to September, infections increased substantially, leading to new government measures on September 28th, intensifying on October 13th, closing restaurants, bars, and theaters, but again keeping stores open and allowing individual use of gyms. On October 13th, the number of cumulative infections since the beginning of the pandemic came to 201 780, while approximately 200 people per day were being hospitalized. The measures partly came as a response to a call from health care workers, indicating they were overwhelmed and regular care was suffering substantially. At the time of writing, it has been announced that measures will continue into January, including restrictions on international travel. Although the relative number of deaths stayed lower in this second wave, an additional 1423 people have died from late July to the first week of November, which was also the time in which our data were collected (all numbers provided by the Dutch government website; <https://www.rivm.nl/coronavirus-covid-19/grafieken>).

**COVID-19 situation in Norway**

As of October 31, 2020, Norway has recorded 20,061 cases and 282 deaths from COVID-19. The pandemic has peaked in two waves to date, the first wave in March, and the second wave beginning in late October. Reported cases per week are going up in Norway as of current writing (October 2020). The number of total reported cases is highest in Oslo (6,391), followed by Bergen (1,914) and Bærum (707). As of current writing, restrictions include no private social gatherings at home with more than 5 guests, no private social gatherings outside home with more than 50 participants, and no public social gatherings with more than 200 participants. Some municipalities take stricter measures depending on the number of reported cases.

**COVID-19 situation in Argentina**

Music & Covid data was collected in Argentina from 4th August to 17th October. During this period, total death count increased from 3,979 to 26,107. Since the beginning of the pandemic the Argentinian government established 5 phases, each one with specific characteristics concerning openings, use of public transport, people movement, etc. that were conveniently administered according to the evolution of the epidemiological situation. The whole Argentine Republic was in phase 1 (full lockdown) since 20th March until 26th April. Since then, an administered lockdown was launched according to the particular COVID-19 transmission in different regions of the country. The metropolitan region (Buenos Aires city and the surrounding area) was affected the most at the beginning, being the situation similar during the first 5 months (March-August). From September onwards the virus spread all over the country (see Figure 1). The delayed virus transmission was a purposeful public governmental policy aimed at (i) preparing the national health system to prevent massive deaths due to lack of intensive care facilities, and (ii) flatten the contagion/death curve.


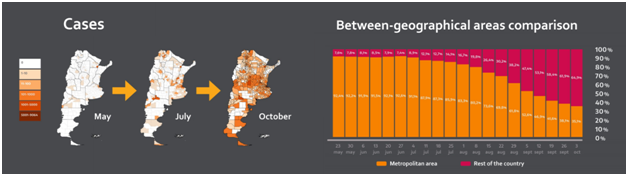


**Figure 1.** Evolution of the pandemic Coronavirus in Argentina.

**COVID-19 situation in Colombia**

On October 16, 2020, Colombia has recorded 945,354 cases and 38,308 deaths from COVID-19, with a cumulative incidence of 1834.53 cases per 100,000 inhabitants and affecting 97.4% of the towns in the country, especially in the main cities: Bogotá D.C., Medellín, Cali, Barranquilla And Cartagena. The average age of active cases is 41.4 years (54%); 78.4% of active cases are at home and 19.5% hospitalized. Between September 13 and October 10, the death rate per 100 000 inhabitants was 8.87. The number of deaths showed increasing variability, from March 16 (1 deceased) to July 30 (358 deaths), from that moment, the number of deaths gradually decreased, until today, October 16, when 173 deceased people appeared. The news indicates that at the end of October there will be a second peak of contagion, although with a lower fatality rate, which is why some cities are once again taking confinement measures, to avoid the collapse of the health system (Ministerio de Salud de Colombia, 2020).

**COVID-19 situation in Mexico**

Mexico is the 13th largest country with 1.973 million km² and the 10th most populated country with over 127,792,286 inhabitants, according to the [Consejo Nacional de Población (CONAPO, 2020)](https://paperpile.com/c/0Yvtvu/7R1i). Unsurprisingly, Mexico has among the highest SARS-CoV-2 cases per capita. However, there is hope as Mexico is the fourth country to adopt the new Pfizer-BioNtech COVID-19 vaccine and it will be accessible to every citizen as of the month of December, beginning with essential health personnel. Mexico City is one of the most densely populated cities. Its geographical conditions, situated in a valley, increases the greenhouse effect making the air pollution constantly putting the city at environmental contingencies and generating pre-existing conditions in its population. The first SARS-CoV-2 case in Mexico was detected in Mexico City on February 28, 2020; two other cases from Culiacán, Sinaloa and Mexico City were reported the same day; those three patients attended a convention in Bergamo, Italy in the third week of February. Since then, Mexico confirmed 1,389,430 positive cases (49.54% women and 50.46% men), 1,746,564 negative cases, 1,014,539 recovered patients, and 122,855 deaths, as of December 27, 2020 [(CONACYT - Dirección General de Epidemiología, 2020)](https://paperpile.com/c/0Yvtvu/wMu41). On March 14, the Ministry of Education suspended in-person classes in elementary and middle schools, moving learning to tele-education. The same day, the Undersecretary of the Ministry of Health announced a series of measures to prevent the spread of the coronavirus in the country. These measures were announced as the “Jornada Nacional de Sana Distancia”, which included the suspension of non-essential economic activities, the restriction of massive congregations, and the recommendation of voluntary home confinement, as well as the Civilian Disaster Relief Plan (Plan DN-III-E) a military operation to carry out relief activities for the society affected by any type of disaster. On March 25 the suspension of all non-essential activities of the Federal Government was informed. Mexico did not implement a mandatory lockdown but promoted voluntary physical distancing and stay-at-home measures. We collected Music & COVID-19 data from the weeks of August 15th (week 33) to November 7th (week 46), 2020, starting four weeks after the first wave peak (week 29) and finishing five weeks before the peak of the second wave (week 51). During this period, Mexico reported 477,336 cases and 46,244 deaths.


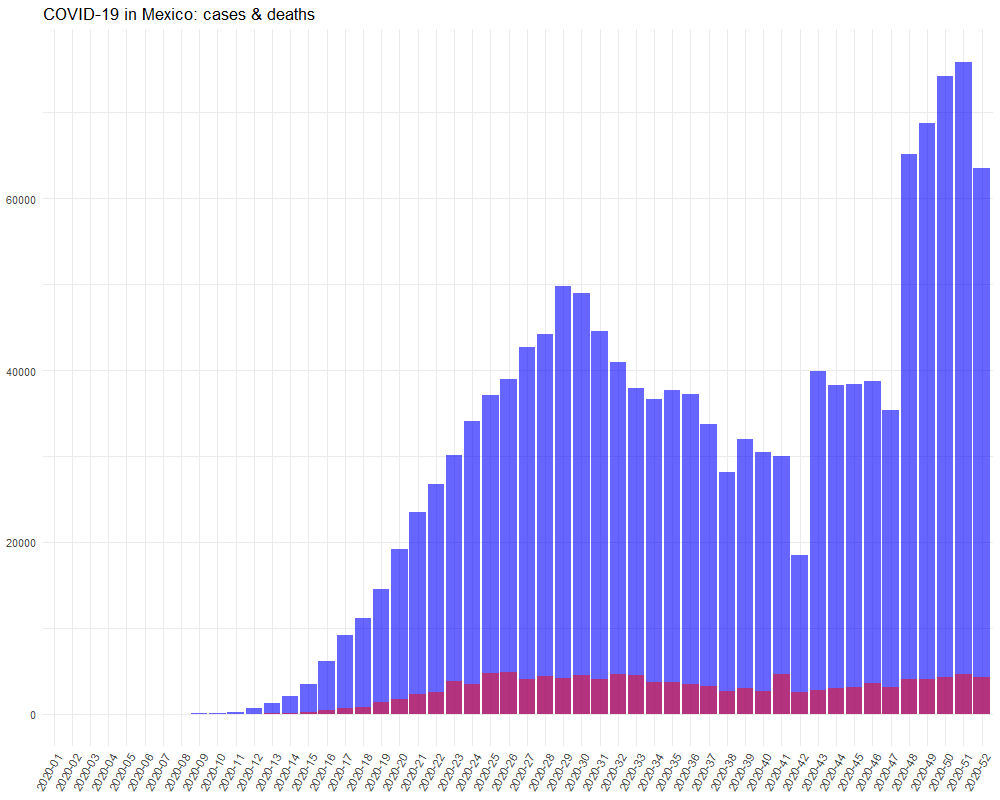


**Figure 3.** Historical data of COVID-19 in Mexico - a weekly reported COVID-19 cases (blue) and deaths (purple) in Mexico during 2020. Based on open data from the European Centre for Disease Prevention and Control (https://opendata.ecdc.europa.eu/).

**COVID-19 situation in Brazil**

Since February, Brazil has been registering an accelerated increase in the number of cases by Covid-19. Important cities like Manaus and São Luís have collapsed their health systems with overcrowded ICUs and the lack of breather machines to help the infected by Coronavirus (Ribeiro, Lima, & Waldman, 2020). Ortega and Orsini (2020) consider that the Brazilian government's actions have been inefficient and contradict the health scientists and the Health World Organization guidelines. Those authors claim that the policy based on science denialism, government by exception, and strategic ignorance have led the country to a health disaster. In October, Brazil registered more than 5 million infected and 150 thousand deaths by this disease (mainly black people and people from the North) (Baqui, MPhil, Marra, Ercole, & Schaar, 2020). The absence of a national plan against the pandemic has led Governors and Mayors to carry out different strategies. Most cities have adopted soft measures such as social distancing, population displacement control, use of face masks, and handwashing (Lima-Costa, Mambrini, Andrade, Peixoto, & Macinko, 2020). Restrictive measures have been created to avoid agglomerations, such as suspension of face-to-face classes (schools and universities), theaters, cinemas, nightclubs, and social and sporting events (Garcia & Duarte, 2020). Bars and restaurants must operate with limited capacity. To reduce the pandemic's economic impact, the Brazilian government has created an Emergency Aid Program that offers an amount of money to the vulnerable population - low income, unemployed, freelance, or microentrepreneur people (approximately 50 million people). Nowadays, scientists have afraid that another wave might make the country's situation worse (Ferrante et al., 2020).

**References**

Baqui, P., MPhil, I., Marra, V., Ercole, A., & Schaar, M. (2020). Ethnic and regional variations in hospital mortality from COVID-19 in Brazil: a cross-sectional observational study. *The Lancet Global Health, 8*(8)*,* e1018-e1026. Doi: [https://doi.org/10.1016/S2214-109X(20)](https://doi.org/10.1016/S2214-109X(20)30285-0)30285-0

Cardoso, B. (2020). The implementation of emergency aid as an exceptional measure of social protection. *Brazilian Journal of Public Administration, 54*(4)*,* 1052-1063. Doi: <https://doi.org/10.1038/s41591-020-1026-x>

[CONACYT - Dirección General de Epidemiología. (2020). COVID-19 Tablero México. Covid-19 México.](http://paperpile.com/b/0Yvtvu/wMu41) <https://datos.covid-19.conacyt.mx/index.php>.

[Consejo Nacional de Población (CONAPO). (2020). Indicadores Demográficos de México de 1950 a 2050.](http://paperpile.com/b/0Yvtvu/7R1i) <http://www.conapo.gob.mx/work/models/CONAPO/Mapa_Ind_Dem18/index_2.html>

Ferrante, L., Steinmetz, W.A., Almeida, A., Leão, J., Vassão, R., Tupinambás, U., Fearnside, P., & Duczmal, L. (2020). Brazil’s policies condemn Amazonia to the second wave of COVID-19. *Nature Medicine, 26***,** 1315. Doi: <https://doi.org/10.1038/s41591-020-1026-x>

Garcia, L. P., & Duarte, E. (2020). Nonpharmaceutical interventions for tackling the COVID-19 epidemic in Brazil. *Epidemiologia e Serviços de Saúde,* *29*(2), e2020222. Doi: <https://doi.org/10.5123/S1679-49742020000200009>

Lima-Costa, M., Mambrini, J., Andrade, F., Peixoto, S., & Macinko, J. (2020). Social distancing, use of face masks and hand washing among participants in the Brazilian Longitudinal Study of Aging: the ELSI-COVID-19 initiative. *Cadernos de Saúde Pública, 36* (Suppl 3), e00193920. Doi: <https://doi.org/10.1590/0102-311X00193920>

Ministerio de salud de Colombia (2020). https://www.minsalud.gov.co/Paginas/Salud-mental-uno-de-los-principales-retos-de-la-pandemia.aspx

Ortega, F., & Orsini, M. (2020). Governing COVID-19 without government in Brazil: Ignorance, neoliberal authoritarianism, and the collapse of public health leadership. *Global Public Health, 15*(9), 1257-1277. <https://doi.org/10.1080/17441692.2020.1795223>

Ribeiro, H., Lima, V., & Waldman, E. (2020). In the COVID-19 pandemic in Brazil, do brown lives matter? *The Lancet Global Health, 8*(8)*,* e-976-e977. <https://doi.org/10.1016/S2214-109X(20)30314-4>

Wu Z, McGoogan JM. Characteristics of and Important Lessons From the Coronavirus Disease 2019 (COVID-19) Outbreak in China: Summary of a Report of 72 314 Cases From the Chinese Center for Disease Control and Prevention. JAMA. 2020; 323(13):1239–1242. <https://doi:10.1001/jama.2020.2648>


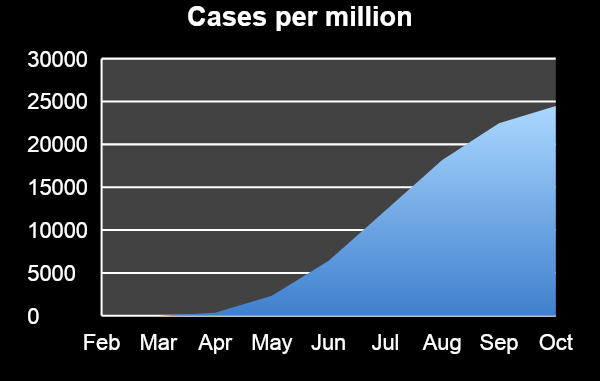

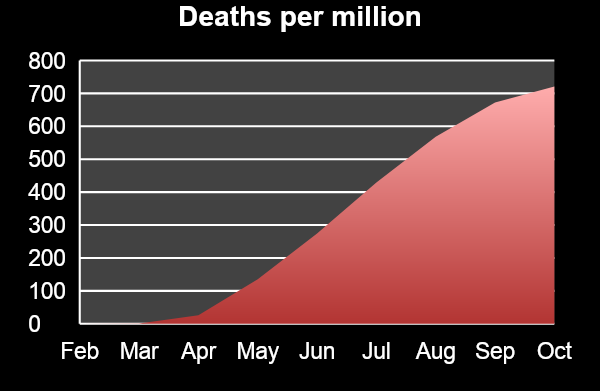


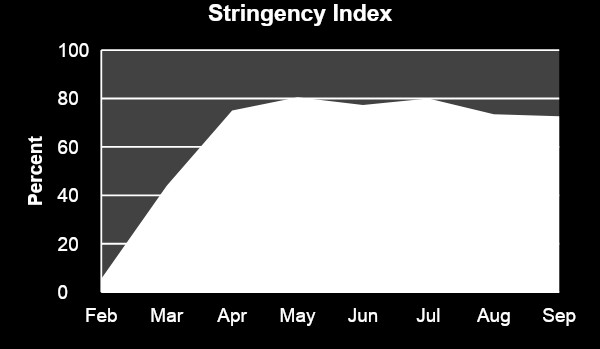


Source: Oxford COVID-19 Government Response Tracker (<https://www.bsg.ox.ac.uk/research/research-projects/coronavirus-government-response-tracker>)

**COVID-19 Timeline:**

1.000 cases (March) 100.000 cases (May)


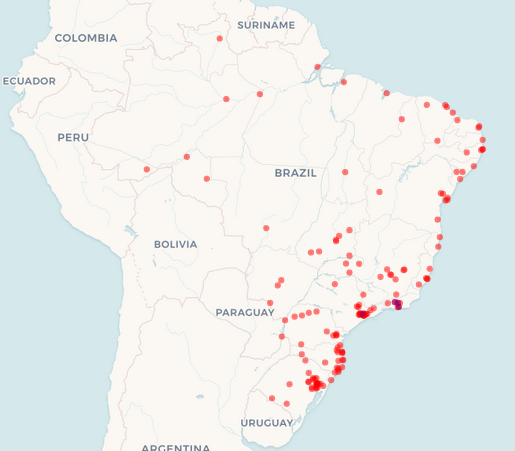

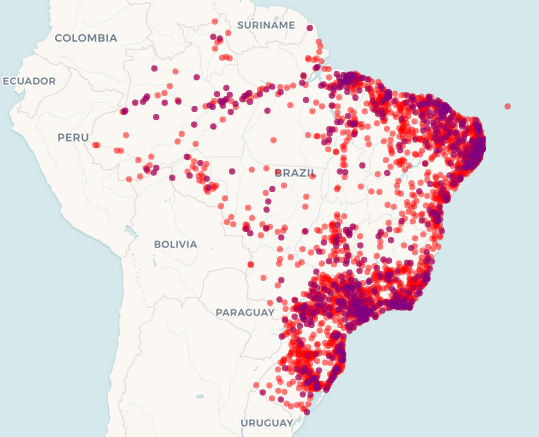


1 million cases (June) 5 million cases (October)


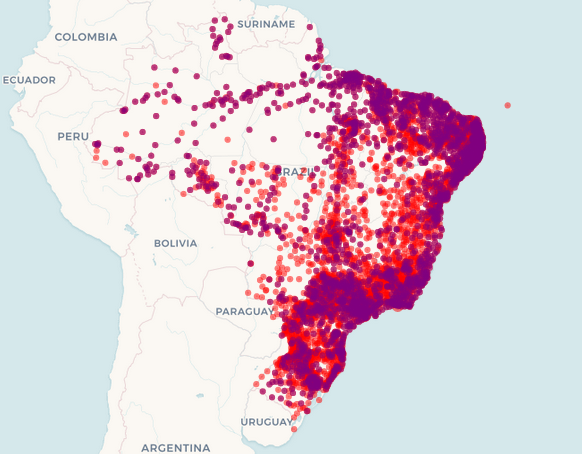

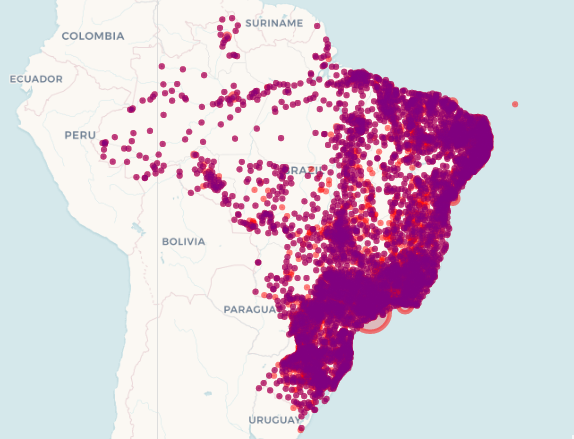


Source: Big-data Covid-19 Fiocruz (https://bigdata-covid19.icict.fiocruz.br/)
