# Supplementary material for: “Help! I Need Somebody”: Music as a Global Resource for Obtaining Wellbeing Goals in Times of Crisis
Source: Front Psychol. 2021 Apr 14;12:648013. doi: 10.3389/fpsyg.2021.648013 (PMC8079817; doi:10.3389/fpsyg.2021.648013)
Supplement: Supplementary file 2 [file Data_Sheet_2.docx]

Appendix 2: Using music during the crisis block

**Using music during the crisis (3-4 min)**

How much is music important to you in general?

- Not at all important (1)
- Slightly important (2)
- Moderately important (3)
- Very important (4)
- Extremely important (5)

How much time did you spend on listening to music during lockdown as compared to the time before the crisis?

- Much less (1)
- Less (2)
- No difference (3)
- More (4)
- Much more (5)

Generally, during the lockdown, the music you have been listening to was:

- Very calming (1)
- Calming (2)
- Slightly calming (3)
- Equally activating and calming (4)
- Slightly activating (5)
- Activating (6)
- Very activating (7)

Generally, during the lockdown, the music you have been listening to was:

- Very optimistic (1)
- Optimistic (2)
- Slightly optimistic (3)
- Equally pessimistic and optimistic (4)
- Slightly pessimistic (5)
- Pessimistic (6)
- Very pessimistic (7)

To what extent have you been listening during the lockdown to music that induces in you feelings of nostalgia?

- Not at all (1)
- A little (2)
- Occasionally (3)
- Often (4)
- A lot (5)
- All the time (6)

During the lockdown have you been listening more to music in English or other language/s?

- Only in English (1)
- Mostly in English (2)
- Equally to both (3)
- Mostly in other language/s (4)
- Only in other language/s (5)

Please write down a song/piece that helped you cope most during the lockdown.

YouTube link or name of song/piece (preferably a link)

________________________________________________________________

Did you participate in weekly clapping for carers and health services?

- Yes, all or most of the time (1)
- Occasionally (2)
- No (3)

How did participating in a weekly clapping for carers and health services affect your sense of togetherness with a larger community?

- Improved it significantly (1)
- Improved moderately (2)
- Did not affect it at all (3)
- Worsened it moderately (4)
- Worsened it significantly (5)

Did you participate in "balcony singing" (singing, playing, banging or hand clapping with neighbors, play music outside, street disco) in your neighborhood? You may pick more than one answer.

- This did not happen/never heard of that (1)
- No, but I would have liked to (2)
- No, and wouldn't want to (3)
- Yes, by playing an instrument (4)
- Yes, by singing (5)
- Yes, by dance or percussion (hand clapping, pan banging, etc.) (6)
- Yes, as a spectator (7)

How did "balcony singing" affect your mood?

- Improved it significantly (1)
- Improved moderately (2)
- Did not affect it at all (3)
- Worsened it moderately (4)
- Worsened it significantly (5)

During the "balcony singing", how much "togetherness" have you been feeling toward other people, in comparison to the rest of the time of the crisis.
If the feeling of "togetherness" wasn't part of your experience, please pick "Irrelevant".

|  | Irrelevant  0 (0) | Much less  1 (1) | Less  2 (2) | The same  3 (3) | More  4 (4) | Much more  5 (5) |
| --- | --- | --- | --- | --- | --- | --- |
| Other participants in the singing (1) |  |  |  |  |  |  |
| People of my neighborhood (2) |  |  |  |  |  |  |
| People of my community (3) |  |  |  |  |  |  |
| People of my city/village (4) |  |  |  |  |  |  |
| People of my country (5) |  |  |  |  |  |  |
| People of the world (6) |  |  |  |  |  |  |

Since the crisis began, how much "togetherness" have you been feeling toward other people?
If the feeling of "togetherness" wasn't part of your experience, please pick "Irrelevant".

|  | Irrelevant  0 (0) | Much less  1 (1) | Less  2 (2) | The same  3 (3) | More  4 (4) | Much more  5 (5) |
| --- | --- | --- | --- | --- | --- | --- |
| People of my neighborhood (2) |  |  |  |  |  |  |
| People of my community (3) |  |  |  |  |  |  |
| People of my city/village (4) |  |  |  |  |  |  |
| People of my country (5) |  |  |  |  |  |  |
| People of the world (6) |  |  |  |  |  |  |

Did you participate in an online joint music playing or singing session (via zoom or Skype etc.)?
If so, how similar is it to a regular face-to-face session?

- I didn't participate (1)
- Absolutely worse (2)
- Somewhat worse (3)
- Different, but with its own benefits (4)
- Very similar (5)
- Much better (6)

What platform or device did you use to listen to music? Choose the option that is most applicable.

- YouTube, other free channels (1)
- Streaming service (e.g. Spotify, iTunes) (2)
- Own collection (CD’s, LP, Tape etc.) (3)
- Radio, TV (4)

Did you make use of **online concerts** as replacements of live events?

- Yes (1)
- No (2)

Did you make use of **online museums** as replacements of live events?

- Yes (1)
- No (2)

Did you make use of **online theater** as replacements of live events?

- Yes (1)
- No (2)
