# Supplementary material for: “Help! I Need Somebody”: Music as a Global Resource for Obtaining Wellbeing Goals in Times of Crisis
Source: Front Psychol. 2021 Apr 14;12:648013. doi: 10.3389/fpsyg.2021.648013 (PMC8079817; doi:10.3389/fpsyg.2021.648013)
Supplement: Supplementary file 3 [file Image_1.pdf]

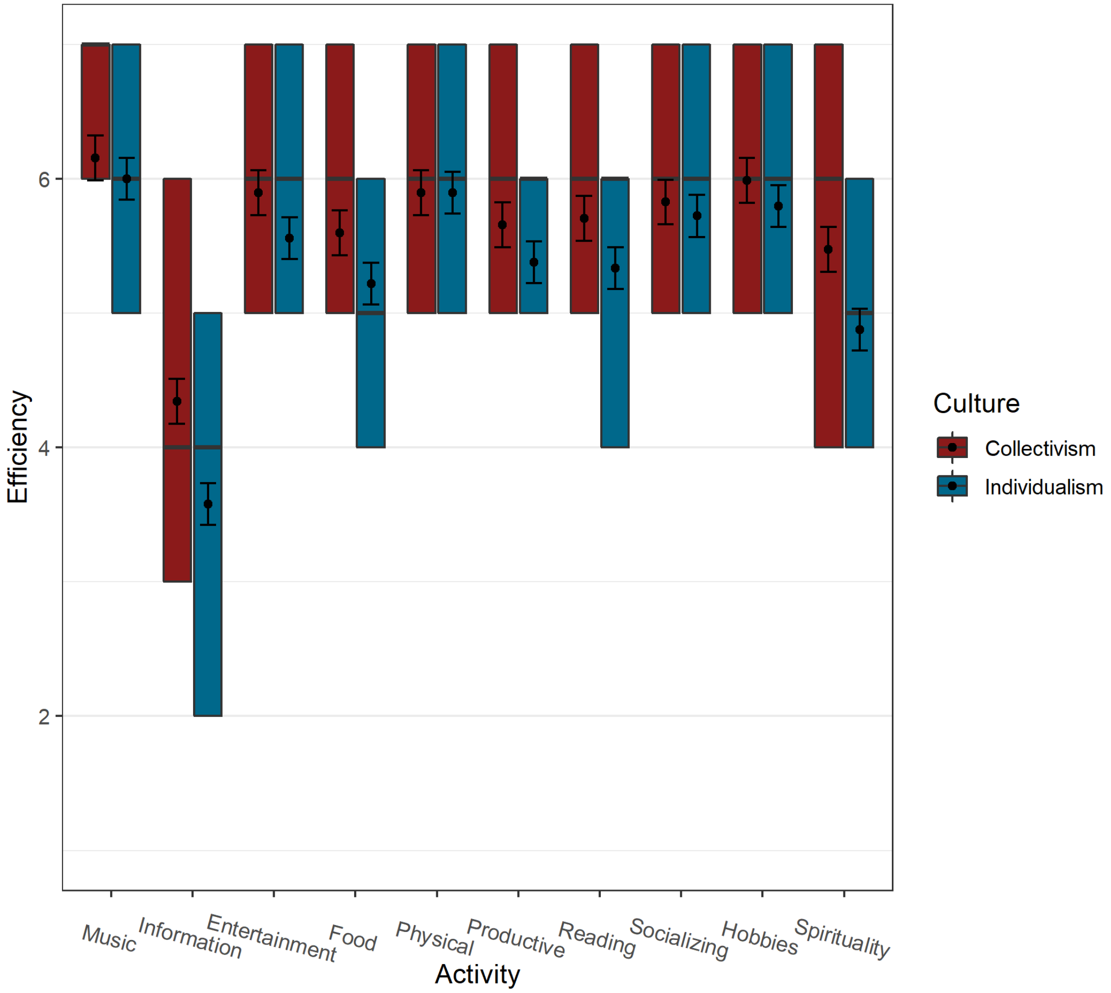

Figure S1. Efficiency of activities in 'Venting negative emotions' by culture. Error bars represent 95% CI Colored squares represent the 2nd and 3rd quartiles of responses. Black lines represent the median, and dots represent the mean.

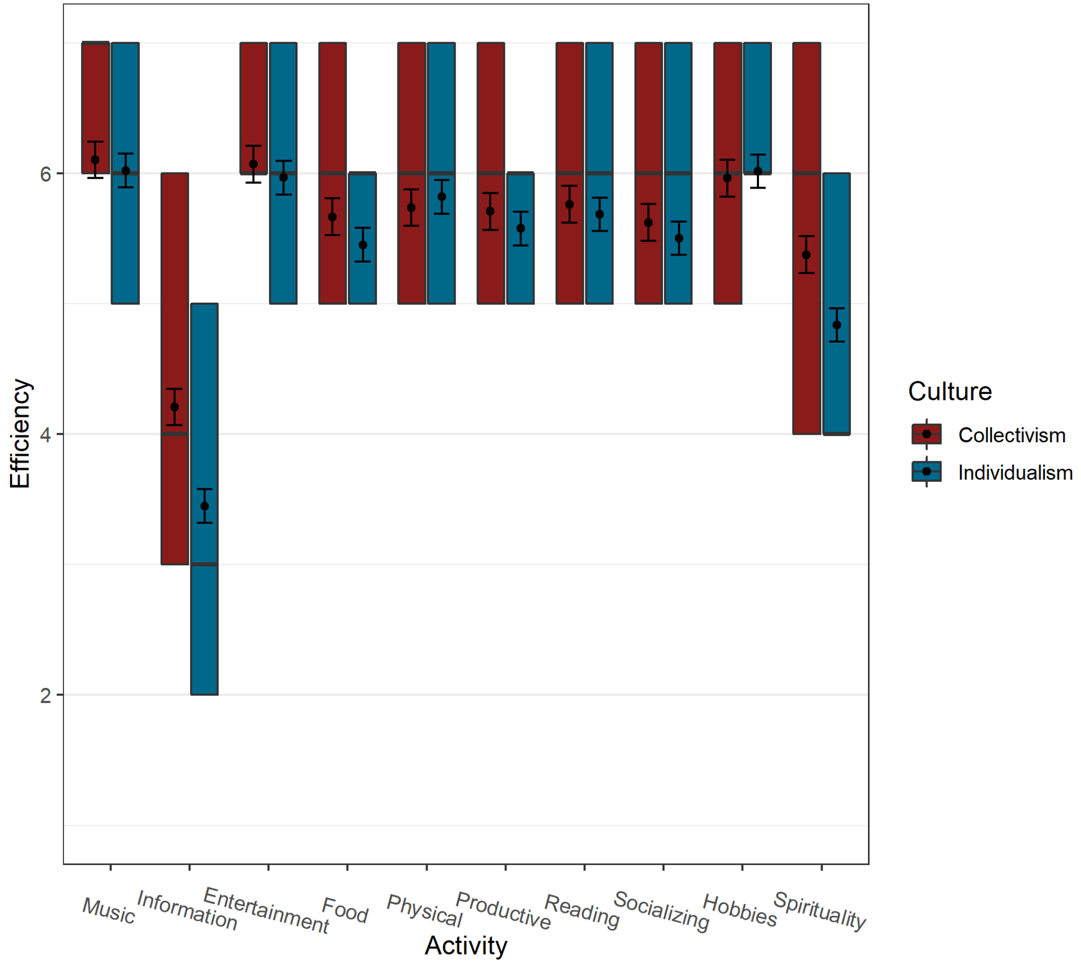

Figure S2. Efficiency of activities in obtaining 'Diversion from the crisis' by culture. Error bars represent 95% CI Colored squares represent the 2nd and 3rd quartiles of responses. Black lines represent the median, and dots represent the mean.

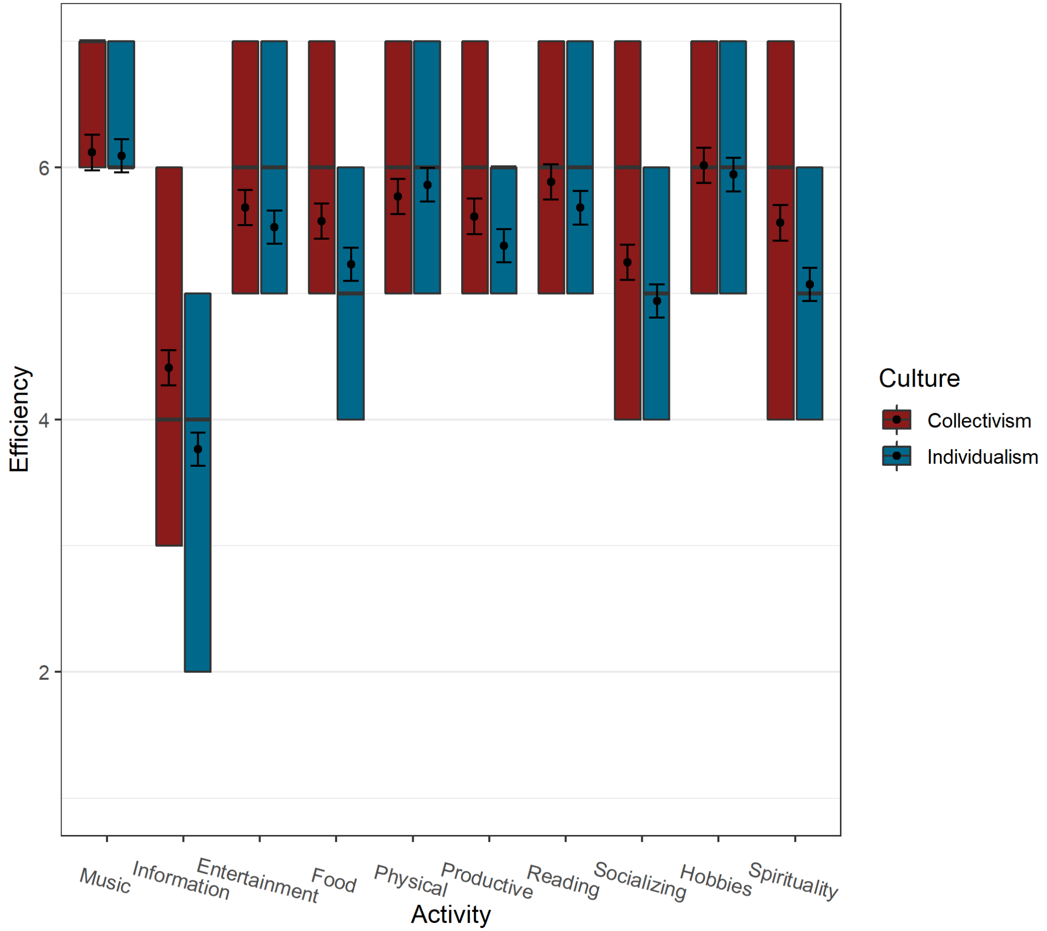

Figure S3. Efficiency of activities in obtaining Self-connection and detachment from the surrounding by culture. Error bars represent 95% CI Colored squares represent the 2nd and 3rd quartiles of responses. Black lines represent the median, and dots represent the mean.

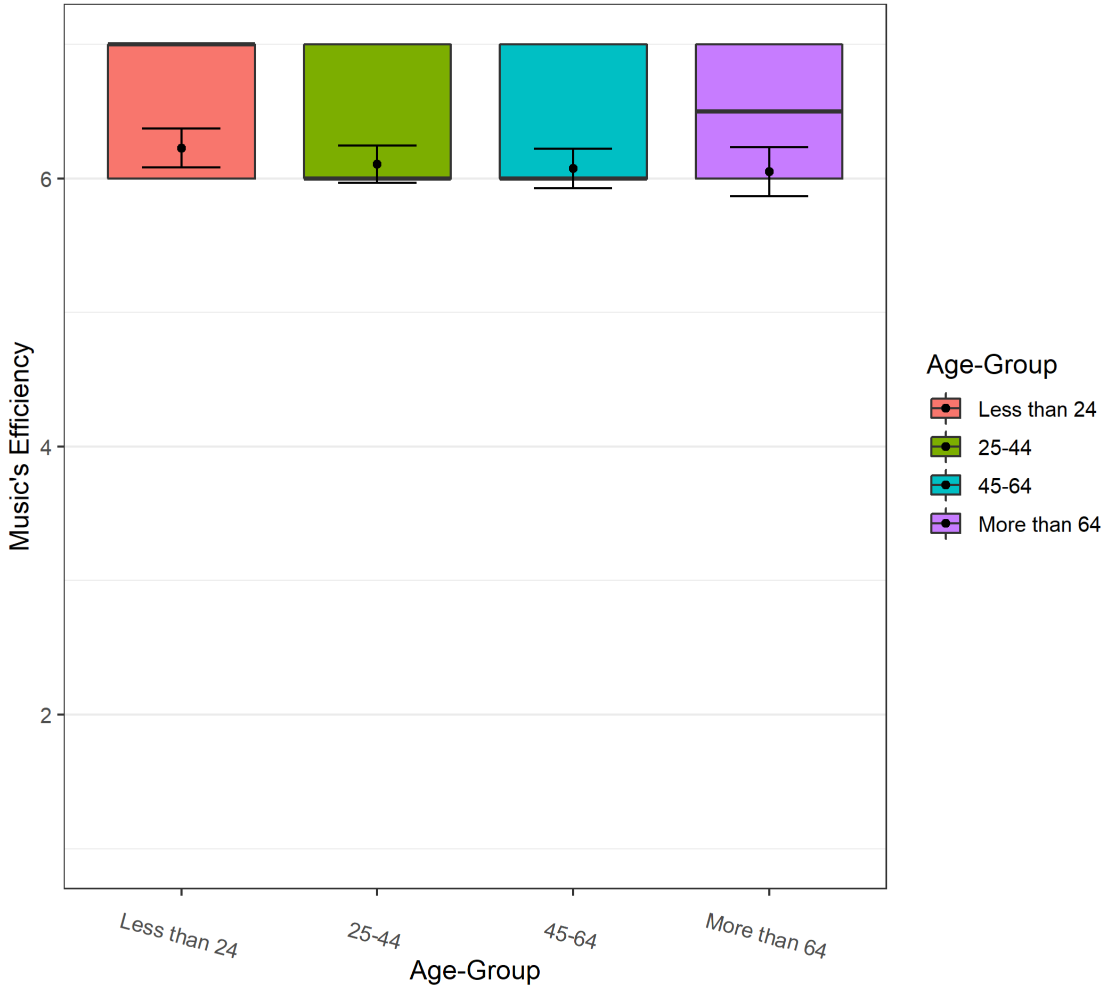

Figure S4. Efficiency of music in obtaining Enjoyment by age. Error bars represent 95% CI Colored squares represent the 2nd and 3rd quartiles of responses. Black lines represent the median, and dots represent the mean.
