# Supplementary material for: “Help! I Need Somebody”: Music as a Global Resource for Obtaining Wellbeing Goals in Times of Crisis
Source: Front Psychol. 2021 Apr 14;12:648013. doi: 10.3389/fpsyg.2021.648013 (PMC8079817; doi:10.3389/fpsyg.2021.648013)
Supplement: Supplementary file 4 [file Table_1.docx]

**Table S1.** Demographic psychological and musical characteristic**s**

| **Variable** | **Value** | **#Total (N=5619)** | **Country** | | | | | | | | | | |
| --- | --- | --- | --- | --- | --- | --- | --- | --- | --- | --- | --- | --- | --- |
|  |  |  | **Argentina**  **(n=588)** | **Brazil (n=470)** | **China (n=443)** | **Colombia (n=901)** | **Italy (n=463)** | **Mexico (n=495)** | **Netherland (n=714)** | **Norway (n=177)** | **Spain (n=507)** | **UK (n=294)** | **USA (n=567)** |
| **Gender** | Male | 1868 | 214 | 129 | 116 | 333 | 145 | 175 | 216 | 83 | 128 | 83 | 246 |
|  | % | 33.46 | 36.52 | 27.68 | 27.04 | 37.12 | 31.32 | 35.50 | 30.42 | 47.16 | 25.45 | 28.23 | 43.54 |
|  | Female | 3615 | 353 | 336 | 302 | 554 | 311 | 310 | 482 | 92 | 369 | 201 | 305 |
|  | % | 64.76 | 60.24 | 72.10 | 70.40 | 61.76 | 67.17 | 62.88 | 67.89 | 52.27 | 73.36 | 68.37 | 53.98 |
|  | Other | 99 | 19 | 1 | 11 | 10 | 7 | 8 | 12 | 1 | 6 | 10 | 14 |
|  | % | 1.77 | 3.24 | 0.21 | 2.56 | 1.11 | 1.51 | 1.62 | 1.69 | 0.57 | 1.19 | 3.40 | 2.48 |
|  | Total | 5582 | 586 | 466 | 429 | 897 | 463 | 493 | 710 | 176 | 503 | 294 | 565 |
| **Age** | Less than 24 | 1479 | 139 | 70 | 223 | 348 | 44 | 123 | 183 | 96 | 69 | 51 | 133 |
|  | % | 26.39 | 23.72 | 14.96 | 50.45 | 38.75 | 9.50 | 24.85 | 25.77 | 54.55 | 13.64 | 17.35 | 23.46 |
|  | 25 – 44 | 2480 | 263 | 262 | 167 | 327 | 306 | 225 | 151 | 57 | 264 | 101 | 357 |
|  | % | 44.25 | 44.88 | 55.98 | 37.78 | 36.41 | 66.09 | 45.45 | 21.27 | 32.39 | 52.17 | 34.35 | 62.96 |
|  | 45 – 64 | 1319 | 151 | 121 | 51 | 201 | 98 | 113 | 247 | 22 | 158 | 94 | 63 |
|  | % | 23.53 | 25.77 | 25.85 | 11.54 | 22.38 | 21.17 | 22.83 | 34.79 | 12.50 | 31.23 | 31.97 | 11.11 |
|  | More than 64 | 327 | 33 | 15 | 1 | 22 | 15 | 34 | 129 | 1 | 15 | 48 | 14 |
|  | % | 5.83 | 5.63 | 3.21 | 0.23 | 2.45 | 3.24 | 6.87 | 18.17 | 0.57 | 2.96 | 16.33 | 2.47 |
|  | Total | 5605 | 586 | 468 | 442 | 898 | 463 | 495 | 710 | 176 | 506 | 294 | 567 |
| **Depression**  **(Range: 0-21)** | Mean | 11.36 | 9.88 | 9.61 | 8.37 | 11.29 | 9.04 | 10.72 | 17.74 | 12.61 | 8.76 | 10.15 | 13.71 |
|  | SD | 11.20 | 9.19 | 8.59 | 9.56 | 11.04 | 8.55 | 10.00 | 15.99 | 10.34 | 8.98 | 8.49 | 11.84 |
|  | Normal | 3968 | 450 | 358 | 359 | 610 | 365 | 366 | 369 | 115 | 404 | 227 | 345 |
|  | % | 71.34 | 77.32 | 76.99 | 82.91 | 69.08 | 78.83 | 74.69 | 52.19 | 64.97 | 80.32 | 77.21 | 61.06 |
|  | Mild | 442 | 54 | 44 | 20 | 67 | 36 | 35 | 60 | 20 | 35 | 27 | 44 |
|  | % | 7.95 | 9.28 | 9.46 | 4.62 | 7.59 | 7.78 | 7.14 | 8.49 | 11.30 | 6.96 | 9.18 | 7.79 |
|  | Moderate | 401 | 30 | 31 | 18 | 76 | 36 | 32 | 50 | 17 | 28 | 20 | 63 |
|  | % | 7.21 | 5.15 | 6.67 | 4.16 | 8.61 | 7.78 | 6.53 | 7.07 | 9.60 | 5.57 | 6.80 | 11.15 |
|  | Severe | 385 | 27 | 22 | 19 | 77 | 17 | 36 | 80 | 14 | 22 | 12 | 59 |
|  | % | 6.92 | 4.64 | 4.73 | 4.39 | 8.72 | 3.67 | 7.35 | 11.32 | 7.91 | 4.37 | 4.08 | 10.44 |
|  | Extremely Severe | 366 | 21 | 10 | 17 | 53 | 9 | 21 | 148 | 11 | 14 | 8 | 54 |
|  | % | 6.58 | 3.61 | 2.15 | 3.93 | 6.00 | 1.94 | 4.29 | 20.93 | 6.21 | 2.78 | 2.72 | 9.56 |
|  | Total | 5562 | 582 | 465 | 433 | 883 | 463 | 490 | 707 | 177 | 503 | 294 | 565 |

| **Variable** | **Value** | **#Total (N=5619)** | **Country** | | | | | | | | | | |
| --- | --- | --- | --- | --- | --- | --- | --- | --- | --- | --- | --- | --- | --- |
|  |  |  | **Argentina**  **(n=588)** | **Brazil (n=470)** | **China (n=443)** | **Colombia (n=901)** | **Italy (n=463)** | **Mexico (n=495)** | **Netherland (n=714)** | **Norway (n=177)** | **Spain (n=507)** | **UK (n=294)** | **USA (n=567)** |
| **Anxiety**  **(Range: 0-21)** | Mean | 8.21 | 6.32 | 7.91 | 8.56 | 8.55 | 4.99 | 7.95 | 12.76 | 6.76 | 6.25 | 5.48 | 10.42 |
|  | SD | 9.68 | 7.35 | 7.95 | 9.53 | 9.45 | 6.31 | 8.52 | 14.39 | 6.69 | 7.51 | 6.74 | 10.64 |
|  | Normal | 3338 | 396 | 264 | 247 | 514 | 340 | 299 | 317 | 110 | 337 | 211 | 303 |
|  | Normal % | 60.00 | 68.28 | 56.77 | 57.44 | 58.21 | 73.59 | 60.53 | 44.90 | 62.15 | 66.60 | 71.77 | 53.53 |
|  | Mild | 429 | 46 | 44 | 34 | 58 | 41 | 33 | 69 | 15 | 41 | 18 | 30 |
|  | Mild % | 7.71 | 7.93 | 9.46 | 7.91 | 6.57 | 8.87 | 6.68 | 9.77 | 8.47 | 8.10 | 6.12 | 5.30 |
|  | Moderate | 758 | 73 | 74 | 73 | 119 | 46 | 82 | 84 | 28 | 64 | 35 | 80 |
|  | Moderate % | 13.63 | 12.59 | 15.91 | 16.98 | 13.48 | 9.96 | 16.60 | 11.90 | 15.82 | 12.65 | 11.90 | 14.13 |
|  | Severe | 298 | 19 | 34 | 19 | 62 | 12 | 24 | 50 | 11 | 27 | 11 | 29 |
|  | Severe % | 5.36 | 3.28 | 7.31 | 4.42 | 7.02 | 2.60 | 4.86 | 7.08 | 6.21 | 5.34 | 3.74 | 5.12 |
|  | Total | 5563 | 580 | 465 | 430 | 883 | 462 | 494 | 706 | 177 | 506 | 294 | 566 |
| **Stress**  **(Range: 0-21)** | Mean | 14.37 | 13.78 | 13.28 | 11.25 | 13.14 | 13.64 | 14.11 | 21.56 | 12.08 | 12.57 | 13.06 | 15.01 |
|  | SD | 11.03 | 8.84 | 8.29 | 9.69 | 10.46 | 8.61 | 9.52 | 16.68 | 8.42 | 8.72 | 8.63 | 10.98 |
|  | Normal | 2002 | 189 | 159 | 210 | 361 | 151 | 164 | 211 | 73 | 186 | 105 | 193 |
|  | % | 36.09 | 32.59 | 34.27 | 48.61 | 40.98 | 32.68 | 33.68 | 29.93 | 41.24 | 37.13 | 35.71 | 34.16 |
|  | Mild | 905 | 122 | 73 | 77 | 126 | 88 | 82 | 64 | 31 | 104 | 59 | 79 |
|  | % | 16.31 | 21.03 | 15.73 | 17.82 | 14.30 | 19.05 | 16.84 | 9.08 | 17.51 | 20.76 | 20.07 | 13.98 |
|  | Moderate | 1350 | 161 | 152 | 78 | 195 | 142 | 142 | 114 | 42 | 118 | 76 | 130 |
|  | % | 24.33 | 27.76 | 32.76 | 18.06 | 22.13 | 30.74 | 29.16 | 16.17 | 23.73 | 23.55 | 25.85 | 23.01 |
|  | Severe | 545 | 53 | 47 | 31 | 84 | 41 | 46 | 61 | 21 | 55 | 32 | 74 |
|  | % | 9.82 | 9.14 | 10.13 | 7.18 | 9.53 | 8.87 | 9.45 | 8.65 | 11.86 | 10.98 | 10.88 | 13.10 |
|  | Extremely Severe | 746 | 55 | 33 | 36 | 115 | 40 | 53 | 255 | 10 | 38 | 22 | 89 |
|  | % | 13.45 | 9.48 | 7.11 | 8.33 | 13.05 | 8.66 | 10.88 | 36.17 | 5.65 | 7.58 | 7.48 | 15.75 |
|  | Total | 5548 | 580 | 464 | 432 | 881 | 462 | 487 | 705 | 177 | 501 | 294 | 565 |
| **Worries related to COVID19**  **(Range: 0-4)** | Mean | 1.77 | 1.66 | 2.36 | 1.39 | 2.06 | 1.59 | 2.11 | 1.15 | 1.71 | 1.85 | 1.56 | 1.92 |
|  | Std. dev. | 0.99 | 0.85 | 0.92 | 1.23 | 1.03 | 0.79 | 0.89 | 0.65 | 0.95 | 0.85 | 0.79 | 1.10 |
|  | Valid N | 5598 | 586 | 466 | 436 | 898 | 462 | 494 | 711 | 177 | 507 | 294 | 567 |
| **Resilience**  **(Range: 0-4)** | Mean | 2.75 | 2.82 | 2.51 | 2.74 | 2.89 | 2.42 | 2.99 | 2.77 | 2.64 | 2.87 | 2.65 | 2.69 |
|  | SD | 0.67 | 0.60 | 0.67 | 0.73 | 0.71 | 0.64 | 0.64 | 0.57 | 0.69 | 0.60 | 0.62 | 0.68 |
|  | Total | 5597 | 584 | 468 | 441 | 895 | 463 | 494 | 710 | 177 | 506 | 293 | 566 |

**Table S1 (cont.)** Musical characteristics

| **Variable** | **Value** | **#Total (N=5619)** | **Country** | | | | | | | | | | |
| --- | --- | --- | --- | --- | --- | --- | --- | --- | --- | --- | --- | --- | --- |
|  |  |  | **Argentina**  **(n=588)** | **Brazil (n=470)** | **China (n=443)** | **Colombia (n=901)** | **Italy (n=463)** | **Mexico (n=495)** | **Netherland (n=714)** | **Norway (n=177)** | **Spain (n=507)** | **UK (n=294)** | **USA (n=567)** |
| **Music's importance (1='Not at all important', 5='Extremely important')** | Mean | 3.98 | 4.17 | 4.40 | 3.34 | 3.81 | 3.76 | 4.19 | 4.16 | 3.79 | 3.95 | 4.14 | 3.94 |
|  | Std. dev. | 1.05 | 0.91 | 0.78 | 1.16 | 1.04 | 1.05 | 0.94 | 0.97 | 1.19 | 1.04 | 1.11 | 1.05 |
|  | Total | 5612 | 586 | 469 | 443 | 900 | 463 | 495 | 712 | 177 | 507 | 293 | 567 |
| **Music-induced nostalgy (1='Not at all', 6='All the time')** | Mean | 2.99 | 2.86 | 3.37 | 3.08 | 2.58 | 2.71 | 2.86 | 3.38 | 3.06 | 2.68 | 2.98 | 3.52 |
|  | Std. dev. | 1.20 | 1.01 | 1.24 | 1.16 | 1.16 | 1.09 | 1.16 | 1.14 | 1.18 | 1.06 | 1.24 | 1.31 |
|  | valid N | 5592 | 583 | 465 | 443 | 896 | 461 | 495 | 711 | 176 | 507 | 292 | 563 |
| **Valence of music (1='Very optimistic', 7='Very pessimistic')** | Mean | 2.98 | 3.13 | 2.51 | 2.79 | 2.81 | 3.31 | 3.02 | 2.75 | 3.67 | 3.11 | 3.28 | 3.09 |
|  | Std. dev. | 1.21 | 1.11 | 1.22 | 1.22 | 1.24 | 1.05 | 1.18 | 1.13 | 1.18 | 1.09 | 1.09 | 1.38 |
|  | Valid N | 5569 | 574 | 468 | 440 | 895 | 457 | 493 | 708 | 177 | 505 | 289 | 563 |
| **Arousal level of music (1='Very calming', 7='Very activating'** | Mean | 4.25 | 4.37 | 4.30 | 4.01 | 4.35 | 4.27 | 4.42 | 4.06 | 4.14 | 4.45 | 3.98 | 4.15 |
|  | Std. dev. | 1.31 | 1.08 | 1.42 | 1.39 | 1.38 | 1.25 | 1.24 | 1.32 | 1.14 | 1.07 | 1.24 | 1.57 |
|  | Valid N | 5590 | 582 | 468 | 443 | 899 | 459 | 495 | 708 | 177 | 506 | 289 | 564 |
| **Experience playing music** | No experience | 2572 | 162 | 254 | 264 | 591 | 256 | 235 | 246 | 68 | 243 | 60 | 193 |
|  | % | 46.13 | 27.79 | 55.34 | 60.55 | 66.03 | 55.41 | 47.57 | 34.70 | 38.64 | 48.31 | 20.48 | 34.10 |
|  | Under 6 year of experience | 1334 | 159 | 81 | 71 | 178 | 99 | 116 | 175 | 67 | 117 | 48 | 223 |
|  | % | 23.92 | 27.27 | 17.65 | 16.28 | 19.89 | 21.43 | 23.48 | 24.68 | 38.07 | 23.26 | 16.38 | 39.40 |
|  | Over 6 year of experience | 1670 | 262 | 124 | 101 | 126 | 107 | 143 | 288 | 41 | 143 | 185 | 150 |
|  | % | 29.95 | 44.94 | 27.02 | 23.17 | 14.08 | 23.16 | 28.95 | 40.62 | 23.30 | 28.43 | 63.14 | 26.50 |
|  | Total | 5576 | 583 | 459 | 436 | 895 | 462 | 494 | 709 | 176 | 503 | 293 | 566 |
| **Balcony singing participation** | No | 4575 | 532 | 337 | 356 | 766 | 312 | 471 | 614 | 163 | 317 | 241 | 466 |
|  | % | 82.49 | 91.88 | 72.94 | 81.09 | 86.46 | 67.68 | 96.12 | 87.46 | 92.09 | 63.40 | 82.82 | 83.36 |
|  | Yes | 971 | 47 | 125 | 83 | 120 | 149 | 19 | 88 | 14 | 183 | 50 | 93 |
|  | % | 17.51 | 8.12 | 27.06 | 18.91 | 13.54 | 32.32 | 3.88 | 12.54 | 7.91 | 36.60 | 17.18 | 16.64 |
|  | Total | 5546 | 579 | 462 | 439 | 886 | 461 | 490 | 702 | 177 | 500 | 291 | 559 |

| **Variable** | **Value** | **#Total (N=5619)** | **Country** | | | | | | | | | | |
| --- | --- | --- | --- | --- | --- | --- | --- | --- | --- | --- | --- | --- | --- |
|  |  |  | **Argentina**  **(n=588)** | **Brazil (n=470)** | **China (n=443)** | **Colombia (n=901)** | **Italy (n=463)** | **Mexico (n=495)** | **Netherland (n=714)** | **Norway (n=177)** | **Spain (n=507)** | **UK (n=294)** | **USA (n=567)** |
| **Platforms for consuming music** | YouTube, other free channels | 2177 | 288 | 242 | 56 | 616 | 174 | 174 | 114 | 24 | 188 | 73 | 228 |
|  | YouTube, other free channels % | 39.13 | 49.74 | 51.71 | 12.81 | 68.75 | 37.99 | 35.29 | 16.19 | 13.56 | 37.38 | 25.7 | 40.35 |
|  | Streaming service (e.g., Spotify, iTunes) | 2514 | 232 | 191 | 344 | 156 | 191 | 266 | 364 | 137 | 235 | 122 | 276 |
|  | Streaming service (e.g., Spotify, iTunes) % | 45.18 | 40.07 | 40.81 | 78.72 | 17.41 | 41.7 | 53.96 | 51.7 | 77.4 | 46.72 | 42.96 | 48.85 |
|  | Own collection (CD’s, LP, Tape etc.) | 474 | 38 | 15 | 16 | 46 | 48 | 39 | 129 | 10 | 46 | 42 | 45 |
|  | Own collection (CD’s, LP, Tape etc.) % | 8.52 | 6.56 | 3.21 | 3.66 | 5.13 | 10.48 | 7.91 | 18.32 | 5.65 | 9.15 | 14.79 | 7.96 |
|  | Radio, TV | 399 | 21 | 20 | 21 | 78 | 45 | 14 | 97 | 6 | 34 | 47 | 16 |
|  | Radio, TV% | 7.17 | 3.63 | 4.27 | 4.81 | 8.71 | 9.83 | 2.84 | 13.78 | 3.39 | 6.76 | 16.55 | 2.83 |
|  | Total valid cases | 5564 | 579 | 468 | 437 | 896 | 458 | 493 | 704 | 177 | 503 | 284 | 565 |
|  | NA's | 55 | 9 | 2 | 6 | 5 | 5 | 2 | 10 | 0 | 4 | 10 | 2 |
| **Participating in a joint Zoom jam session** | I didn't participate | 3775 | 310 | 308 | 265 | 627 | 370 | 301 | 540 | 147 | 335 | 163 | 409 |
|  | I didn't participate % | 68.64 | 54.96 | 67.99 | 60.23 | 70.69 | 80.43 | 62.06 | 77.59 | 83.52 | 69.07 | 56.01 | 72.65 |
|  | Absolutely worse | 360 | 89 | 11 | 9 | 39 | 15 | 37 | 66 | 6 | 31 | 38 | 19 |
|  | Absolutely worse % | 6.55 | 15.78 | 2.43 | 2.05 | 4.4 | 3.26 | 7.63 | 9.48 | 3.41 | 6.39 | 13.06 | 3.37 |
|  | Somewhat worse | 302 | 75 | 13 | 16 | 29 | 13 | 35 | 29 | 13 | 35 | 23 | 21 |
|  | Somewhat worse% | 5.49 | 13.3 | 2.87 | 3.64 | 3.27 | 2.83 | 7.22 | 4.17 | 7.39 | 7.22 | 7.9 | 3.73 |
|  | Different, but with its own benefits | 871 | 84 | 89 | 122 | 161 | 50 | 103 | 58 | 8 | 72 | 66 | 58 |
|  | Different, but with its own benefits % | 15.84 | 14.89 | 19.65 | 27.73 | 18.15 | 10.87 | 21.24 | 8.33 | 4.55 | 14.85 | 22.68 | 10.3 |
|  | Very similar | 110 | 3 | 15 | 11 | 24 | 9 | 5 | 1 | 2 | 6 | - | 34 |
|  | Very similar% | 2 | 0.53 | 3.31 | 2.5 | 2.71 | 1.96 | 1.03 | 0.14 | 1.14 | 1.24 | - | 6.04 |
|  | Much better | 82 | 3 | 17 | 17 | 7 | 3 | 4 | 2 | - | 6 | 1 | 22 |
|  | Much better % | 1.49 | 0.53 | 3.75 | 3.86 | 0.79 | 0.65 | 0.82 | 0.29 | - | 1.24 | 0.34 | 3.91 |
|  | Total valid cases | 5500 | 564 | 453 | 440 | 887 | 460 | 485 | 696 | 176 | 485 | 291 | 563 |
|  | NA's | 119 | 24 | 17 | 3 | 14 | 3 | 10 | 18 | 1 | 22 | 3 | 4 |

**Table S2.** Overview of main statistical outcomes of multilevel regression models testing the efficiency of activities in obtaining ‘Venting negative emotions' by Activity and Culture.

|  | **Model-0** | | **Model-1** | | **Model-2** | | **Model-3** | |
| --- | --- | --- | --- | --- | --- | --- | --- | --- |
|  | *Estimates* | *CI* | *Estimates* | *CI* | *Estimates* | *CI* | *Estimates* | *CI* |
| **Intercept** | 5.48 ^***^ | 5.34 – 5.62 | 6.06 ^***^ | 5.91 – 6.21 | 6.23 ^***^ | 6.07 – 6.40 | 6.16 ^***^ | 5.99 – 6.32 |
| **Activity** |  |  |  |  |  |  |  |  |
| Information Seeking |  |  | -2.11 ^***^ | -2.16 – -2.06 | -2.11 ^***^ | -2.16 – -2.06 | -1.81 ^***^ | -1.88 – -1.74 |
| Entertainment |  |  | -0.35 ^***^ | -0.40 – -0.30 | -0.35 ^***^ | -0.40 – -0.30 | -0.26 ^***^ | -0.33 – -0.19 |
| Food |  |  | -0.67 ^***^ | -0.72 – -0.62 | -0.67 ^***^ | -0.72 – -0.62 | -0.56 ^***^ | -0.63 – -0.49 |
| Physical |  |  | -0.18 ^***^ | -0.23 – -0.13 | -0.18 ^***^ | -0.23 – -0.13 | -0.26 ^***^ | -0.33 – -0.19 |
| Productive |  |  | -0.56 ^***^ | -0.61 – -0.51 | -0.56 ^***^ | -0.61 – -0.51 | -0.50 ^***^ | -0.57 – -0.43 |
| Reading |  |  | -0.55 ^***^ | -0.60 – -0.50 | -0.55 ^***^ | -0.60 – -0.50 | -0.45 ^***^ | -0.52 – -0.38 |
| Socializing |  |  | -0.30 ^***^ | -0.35 – -0.25 | -0.30 ^***^ | -0.35 – -0.25 | -0.33 ^***^ | -0.40 – -0.26 |
| Hobbies |  |  | -0.18 ^***^ | -0.23 – -0.13 | -0.18 ^***^ | -0.23 – -0.13 | -0.17 ^***^ | -0.24 – -0.10 |
| Spirituality |  |  | -0.89 ^***^ | -0.94 – -0.84 | -0.89 ^***^ | -0.94 – -0.84 | -0.68 ^***^ | -0.75 – -0.61 |
| **Culture dummy** |  |  |  |  | -0.32 ^**^ | -0.54 – -0.10 | -0.16 | -0.38 – 0.07 |
| **Activity × Culture** |  |  |  |  |  |  |  |  |
| Information × Culture |  |  |  |  |  |  | -0.61 ^***^ | -0.71 – -0.51 |
| Entertainment × Culture |  |  |  |  |  |  | -0.18 ^***^ | -0.28 – -0.08 |
| Food × Culture |  |  |  |  |  |  | -0.22 ^***^ | -0.32 – -0.12 |
| Physical × Culture |  |  |  |  |  |  | 0.16 ^**^ | 0.06 – 0.26 |
| Productive × Culture |  |  |  |  |  |  | -0.12 ^*^ | -0.22 – -0.02 |
| Reading × Culture |  |  |  |  |  |  | -0.21 ^***^ | -0.31 – -0.11 |
| Socializing × Culture |  |  |  |  |  |  | 0.05 | -0.05 – 0.15 |
| Hobbies × Culture |  |  |  |  |  |  | -0.04 | -0.13 – 0.06 |
| Spirituality × Culture |  |  |  |  |  |  | -0.44 ^***^ | -0.54 – -0.34 |
| **Random Effects** |  |  |  |  |  |  |  |  |
| σ^2^ | 1.69 |  | 1.33 |  | 1.33 |  | 1.32 |  |
| τ_00_ _respondent_ | 0.30 |  | 0.34 |  | 0.34 |  | 0.34 |  |
| _country_ | 0.06 |  | 0.06 |  | 0.03 |  | 0.03 |  |
| N _respondent_ | 4109 |  | 4109 |  | 4109 |  | 4109 |  |
| _country_ | 11 |  | 11 |  | 11 |  | 11 |  |
| ICC _respondent_ | 0.147 |  |  |  |  |  |  |  |
| _country_ | 0.028 |  |  |  |  |  |  |  |
| Observations | 40749 |  | 40749 |  | 40749 |  | 40749 |  |
| Marginal R^2^ / Conditional R^2^ | 0.000 / 0.175 |  | 0.157 / 0.350 |  | 0.169 / 0.350 |  | 0.175 / 0.356 |  |

Note: Culture dummy = 1 for individualistic culture, 0 for collectivistic culture. Reference Activity is Music.

* *p*<0.05   ** *p*<0.01   *** *p*<0.001

**Table S3.** Overview of main statistical outcomes of multilevel regression models testing the efficiency of activities in obtaining ‘Diversion from the crisis' by Activity and Culture.

|  | **Model-0** | | **Model-1** | | **Model-2** | | **Model-3** | |
| --- | --- | --- | --- | --- | --- | --- | --- | --- |
|  | *Estimates* | *CI* | *Estimates* | *CI* | *Estimates* | *CI* | *Estimates* | *CI* |
| **Intercept** | 5.52 *** | 5.42 – 5.62 | 6.06 *** |  | 6.16 *** | 6.03 – 6.30 | 6.10 *** | 5.96 – 6.24 |
| **Activity** |  |  |  |  |  |  |  |  |
| Information Seeking |  |  | -2.27 *** | -2.32 – -2.21 | -2.27 *** | -2.32 – -2.21 | -1.90 *** | -1.98 – -1.82 |
| Entertainment |  |  | -0.04 | -0.10 – 0.01 | -0.04 | -0.10 – 0.01 | -0.03 | -0.11 – 0.05 |
| Food |  |  | -0.51 *** | -0.56 – -0.46 | -0.51 *** | -0.56 – -0.46 | -0.44 *** | -0.52 – -0.36 |
| Physical |  |  | -0.28 *** | -0.33 – -0.22 | -0.28 *** | -0.33 – -0.22 | -0.37 *** | -0.45 – -0.29 |
| Productive |  |  | -0.42 *** | -0.48 – -0.37 | -0.42 *** | -0.48 – -0.37 | -0.40 *** | -0.47 – -0.32 |
| Reading |  |  | -0.34 *** | -0.39 – -0.28 | -0.34 *** | -0.39 – -0.28 | -0.34 *** | -0.42 – -0.26 |
| Socializing |  |  | -0.50 *** | -0.56 – -0.45 | -0.50 *** | -0.56 – -0.45 | -0.48 *** | -0.56 – -0.40 |
| Hobbies |  |  | -0.07 * | -0.12 – -0.01 | -0.07 * | -0.12 – -0.01 | -0.14 *** | -0.22 – -0.06 |
| Spirituality |  |  | -0.98 *** | -1.03 – -0.92 | -0.98 *** | -1.03 – -0.92 | -0.73 *** | -0.81 – -0.65 |
| **Culture dummy** |  |  |  |  | -0.19 * | -0.37 – -0.01 | -0.08 | -0.27 – 0.11 |
| **Activity × Culture** |  |  |  |  |  |  |  |  |
| Information × Culture |  |  |  |  |  |  | -0.68 *** | -0.78 – -0.57 |
| Entertainment × Culture |  |  |  |  |  |  | -0.02 | -0.13 – 0.09 |
| Food × Culture |  |  |  |  |  |  | -0.13 * | -0.24 – -0.03 |
| Physical × Culture |  |  |  |  |  |  | 0.16 ** | 0.06 – 0.27 |
| Productive × Culture |  |  |  |  |  |  | -0.05 | -0.16 – 0.06 |
| Reading × Culture |  |  |  |  |  |  | 0.00 | -0.10 – 0.11 |
| Socializing × Culture |  |  |  |  |  |  | -0.04 | -0.15 – 0.07 |
| Hobbies × Culture |  |  |  |  |  |  | 0.14 * | 0.03 – 0.24 |
| Spirituality × Culture |  |  |  |  |  |  | -0.46 *** | -0.56 – -0.35 |
| **Random Effects** |  |  |  |  |  |  |  |  |
| σ^2^ | 1.81 |  | 1.36 |  | 1.36 |  | 1.35 |  |
| τ_00_ _respondent_ | 0.29 |  | 0.34 |  | 0.34 |  | 0.34 |  |
| _country_ | 0.03 |  | 0.03 |  | 0.02 |  | 0.02 |  |
| N _respondent_ | 3636 |  | 3636 |  | 3636 |  | 3636 |  |
| _country_ | 11 |  | 11 |  | 11 |  | 11 |  |
| ICC _respondent_ | 0.138 |  |  |  |  |  |  |  |
| _country_ | 0.013 |  |  |  |  |  |  |  |
| Observations | 36028 |  | 36028 |  | 36028 |  | 36028 |  |
| Marginal R^2^ / Conditional R^2^ | 0.000 / 0.151 |  | 0.190 / 0.362 |  | 0.194 / 0.363 |  | 0.201 / 0.371 |  |

Note: Culture dummy = 1 for individualistic culture, 0 for collectivistic culture. Reference Activity is Music

* *p*<0.05   *** *p*<0.001**Table S4.** Overview of main statistical outcomes of multilevel regression models testing the efficiency of activities in obtaining ‘Self-connection and detachment from the surrounding' by Activity and Culture.

|  | **Model-0** | | **Model-1** | | **Model-2** | | **Model-3** | |
| --- | --- | --- | --- | --- | --- | --- | --- | --- |
|  | *Estimates* | *CI* | *Estimates* | *CI* | *Estimates* | *CI* | *Estimates* | *CI* |
| **Intercept** | 5.46 *** | 5.34 – 5.57 | 6.09 *** | 5.97 – 6.21 | 6.22 *** | 6.08 – 6.35 | 6.12 *** | 5.98 – 6.26 |
| **Activity** |  |  |  |  |  |  |  |  |
| Information Seeking |  |  | -2.00 *** | -2.05 – -1.95 | -2.00 *** | -2.05 – -1.95 | -1.71 *** | -1.78 – -1.63 |
| Entertainment |  |  | -0.50 *** | -0.55 – -0.44 | -0.50 *** | -0.55 – -0.44 | -0.44 *** | -0.51 – -0.36 |
| Food |  |  | -0.69 *** | -0.75 – -0.64 | -0.69 *** | -0.75 – -0.64 | -0.55 *** | -0.62 – -0.47 |
| Physical |  |  | -0.29 *** | -0.34 – -0.24 | -0.29 *** | -0.34 – -0.24 | -0.35 *** | -0.42 – -0.28 |
| Productive |  |  | -0.60 *** | -0.66 – -0.55 | -0.60 *** | -0.66 – -0.55 | -0.51 *** | -0.58 – -0.43 |
| Reading |  |  | -0.32 *** | -0.37 – -0.26 | -0.32 *** | -0.37 – -0.26 | -0.23 *** | -0.30 – -0.16 |
| Socializing |  |  | -1.00 *** | -1.06 – -0.95 | -1.00 *** | -1.06 – -0.95 | -0.87 *** | -0.94 – -0.80 |
| Hobbies |  |  | -0.12 *** | -0.18 – -0.07 | -0.12 *** | -0.18 – -0.07 | -0.10 ** | -0.17 – -0.03 |
| Spirituality |  |  | -0.78 *** | -0.83 – -0.72 | -0.78 *** | -0.83 – -0.72 | -0.56 *** | -0.63 – -0.49 |
| **Culture dummy** |  |  |  |  | -0.24 ** | -0.42 – -0.06 | -0.03 | -0.22 – 0.17 |
| **Activity × Culture** |  |  |  |  |  |  |  |  |
| Information × Culture |  |  |  |  |  |  | -0.62 *** | -0.72 – -0.51 |
| Entertainment × Culture |  |  |  |  |  |  | -0.13 * | -0.24 – -0.03 |
| Food × Culture |  |  |  |  |  |  | -0.32 *** | -0.42 – -0.21 |
| Physical × Culture |  |  |  |  |  |  | 0.12 * | 0.01 – 0.22 |
| Productive × Culture |  |  |  |  |  |  | -0.21 *** | -0.31 – -0.10 |
| Reading × Culture |  |  |  |  |  |  | -0.18 *** | -0.28 – -0.07 |
| Socializing × Culture |  |  |  |  |  |  | -0.28 *** | -0.39 – -0.18 |
| Hobbies × Culture |  |  |  |  |  |  | -0.05 | -0.15 – 0.06 |
| Spirituality × Culture |  |  |  |  |  |  | -0.46 *** | -0.57 – -0.36 |
| **Random Effects** |  |  |  |  |  |  |  |  |
| σ^2^ | 1.74 |  | 1.41 |  | 1.41 |  | 1.40 |  |
| τ_00_ _respondent_ | 0.33 |  | 0.37 |  | 0.37 |  | 0.37 |  |
| _country_ | 0.03 |  | 0.03 |  | 0.02 |  | 0.02 |  |
| N _respondent_ | 3971 |  | 3971 |  | 3971 |  | 3971 |  |
| _country_ | 11 |  | 11 |  | 11 |  | 11 |  |
| ICC _respondent_ | 0.159 |  |  |  |  |  |  |  |
| _country_ | 0.016 |  |  |  |  |  |  |  |
| Observations | 39358 |  | 39358 |  | 39358 |  | 39358 |  |
| Marginal R^2^ / Conditional R^2^ | 0.000 / 0.175 |  | 0.139 / 0.329 |  | 0.145 / 0.330 |  | 0.150 / 0.335 |  |

Note: Culture dummy = 1 for individualistic culture, 0 for collectivistic culture. Reference Activity is Music

* *p*<0.05   ** *p*<0.01   *** *p*<0.001

**Table S5.** Overview of main statistical outcomes of multilevel regression models testing which variables predict the rated efficiency of music in obtaining ‘Diversion from the Crisis'.

|  | **Model-0** | | **Model-1** | | **Model-2** | | **Model-3** | | **Model-4** | | **Model-5** | |
| --- | --- | --- | --- | --- | --- | --- | --- | --- | --- | --- | --- | --- |
|  | *Estimates* | *std. Error* | *Estimates* | *std. Error* | *Estimates* | *std. Error* | *Estimates* | *std. Error* | *Estimates* | *std. Error* | *Estimates* | *std. Error* |
| **Intercept** | -0.00 | 0.06 | 0.08 | 0.10 | -0.03 | 0.10 | -0.01 | 0.09 | -0.02 | 0.08 | -0.07 | 0.06 |
| **Age** |  |  |  |  |  |  |  |  |  |  |  |  |
| 25-44 |  |  | -0.10 ^*^ | 0.04 | -0.10 ^*^ | 0.04 | -0.11 ^**^ | 0.04 | -0.11 ^*^ | 0.04 | -0.05 | 0.04 |
| 45-64 |  |  | -0.03 | 0.05 | -0.01 | 0.05 | -0.03 | 0.05 | -0.03 | 0.05 | 0.01 | 0.04 |
| More than 64 |  |  | -0.06 | 0.08 | -0.04 | 0.08 | -0.04 | 0.08 | -0.06 | 0.08 | 0.06 | 0.07 |
| **Female dummy** |  |  | 0.02 | 0.04 | 0.02 | 0.04 | 0.01 | 0.04 | 0.01 | 0.04 | 0.04 | 0.03 |
| **Culture dummy** |  |  | -0.07 | 0.13 | -0.09 | 0.12 | -0.09 | 0.11 | -0.07 | 0.09 | -0.04 | 0.07 |
| **Goal importance** |  |  |  |  | 0.20 ^***^ | 0.03 | 0.19 ^***^ | 0.03 | 0.17 ^***^ | 0.03 | 0.09 ^***^ | 0.02 |
| **Openness to experience** |  |  |  |  |  |  | 0.11 ^***^ | 0.02 | 0.11 ^***^ | 0.02 | 0.04 ^**^ | 0.02 |
| **Valence of music** |  |  |  |  |  |  |  |  | -0.11 ^***^ | 0.02 | -0.09 ^***^ | 0.02 |
| **Music-induced nostalgia** |  |  |  |  |  |  |  |  | 0.15 ^***^ | 0.02 | 0.04 ^**^ | 0.02 |
| **Music's importance** |  |  |  |  |  |  |  |  |  |  | 0.47 ^***^ | 0.02 |
| **Random Effects** |  |  |  |  |  |  |  |  |  |  |  |  |
| σ^2^ | 0.96 |  | 0.96 |  | 0.95 |  | 0.94 |  | 0.91 |  | 0.74 |  |
| τ_00_ _country_ | 0.04 |  | 0.04 |  | 0.04 |  | 0.03 |  | 0.02 |  | 0.01 |  |
| ICC _country_ | 0.04 |  |  |  |  |  |  |  |  |  |  |  |
| N _country_ | 11 |  | 11 |  | 11 |  | 11 |  | 11 |  | 11 |  |
| Observations | 3467 |  | 3467 |  | 3467 |  | 3467 |  | 3467 |  | 3467 |  |
| Marginal R^2^ / Conditional R^2^ | 0.000 / 0.037 |  | 0.003 / 0.044 |  | 0.018 / 0.055 |  | 0.030 / 0.060 |  | 0.060 / 0.081 |  | 0.250 / 0.261 |  |

Note: Culture dummy = 1 for individualistic culture, 0 for collectivistic culture. Reference age is less than 24.

Female dummy = 1 for female, 0 for males.

* *p*<0.05   ** *p*<0.01   *** *p*<0.001

**Table S6.** Overview of main statistical outcomes of multilevel regression models testing which variables predict the rated efficiency of music in obtaining ‘Enjoyment and maintaining good mood'.

|  | **Model-0** | | **Model-1** | | **Model-2** | | **Model-3** | | **Model-4** | | **Model-5** | |
| --- | --- | --- | --- | --- | --- | --- | --- | --- | --- | --- | --- | --- |
|  | *Estimates* | *std. Error* | *Estimates* | *std. Error* | *Estimates* | *std. Error* | *Estimates* | *std. Error* | *Estimates* | *std. Error* | *Estimates* | *std. Error* |
| **Intercept** | -0.01 | 0.06 | 0.11 | 0.10 | 0.07 | 0.09 | 0.08 | 0.08 | 0.06 | 0.06 | -0.02 | 0.05 |
| **Age** |  |  |  |  |  |  |  |  |  |  |  |  |
| 25-44 |  |  | -0.10 ^**^ | 0.04 | -0.10 ^**^ | 0.04 | -0.11 ^**^ | 0.04 | -0.09 ^*^ | 0.04 | -0.04 | 0.03 |
| 45-64 |  |  | -0.13 ^**^ | 0.04 | -0.12 ^**^ | 0.04 | -0.15 ^***^ | 0.04 | -0.10 ^*^ | 0.04 | -0.04 | 0.04 |
| More than 64 |  |  | -0.17 ^*^ | 0.07 | -0.16 ^*^ | 0.07 | -0.18 ^**^ | 0.07 | -0.16 ^*^ | 0.07 | -0.04 | 0.06 |
| **Female dummy** |  |  | 0.00 | 0.03 | -0.00 | 0.03 | -0.00 | 0.03 | 0.00 | 0.03 | 0.03 | 0.03 |
| **Culture dummy** |  |  | -0.08 | 0.13 | -0.06 | 0.11 | -0.06 | 0.10 | -0.05 | 0.08 | -0.04 | 0.06 |
| **Goal importance** |  |  |  |  | 0.23 ^***^ | 0.02 | 0.20 ^***^ | 0.02 | 0.19 ^***^ | 0.02 | 0.13 ^***^ | 0.02 |
| **Openness to experience** |  |  |  |  |  |  | 0.14 ^***^ | 0.01 | 0.14 ^***^ | 0.01 | 0.07 ^***^ | 0.01 |
| **Valence of music** |  |  |  |  |  |  |  |  | -0.06 ^***^ | 0.01 | -0.06 ^***^ | 0.01 |
| **Music-induced nostalgia** |  |  |  |  |  |  |  |  | 0.15 ^***^ | 0.01 | 0.06 ^***^ | 0.01 |
| **Music's importance** |  |  |  |  |  |  |  |  |  |  | 0.47 ^***^ | 0.01 |
|  |  |  |  |  |  |  |  |  |  |  |  |  |
| **Random Effects** |  |  |  |  |  |  |  |  |  |  |  |  |
| σ^2^ | 0.95 |  | 0.95 |  | 0.93 |  | 0.91 |  | 0.89 |  | 0.71 |  |
| τ_00_ _country_ | 0.04 |  | 0.04 |  | 0.03 |  | 0.02 |  | 0.01 |  | 0.01 |  |
| ICC _country_ | 0.04 |  |  |  |  |  |  |  |  |  |  |  |
| N _country_ | 11 |  | 11 |  | 11 |  | 11 |  | 11 |  | 11 |  |
| Observations | 4697 |  | 4697 |  | 4697 |  | 4697 |  | 4697 |  | 4697 |  |
| Marginal R^2^ / Conditional R^2^ | 0.000 / 0.038 |  | 0.005 / 0.049 |  | 0.033 / 0.063 |  | 0.054 / 0.077 |  | 0.080 / 0.094 |  | 0.267 / 0.274 |  |

Note: Culture dummy = 1 for individualistic culture, 0 for collectivistic culture. Reference age is less than 24.

Female dummy = 1 for female, 0 for males.

* *p*<0.05   ** *p*<0.01   *** *p*<0.001

**Table S7.** Overview of main statistical outcomes of multilevel regression models testing which variables predict the rated efficiency of music in ‘Reducing loneliness and creating a sense of togetherness'.

|  | **Model-0** | | **Model-1** | | **Model-2** | | **Model-3** | | **Model-4** | | **Model-5** | |
| --- | --- | --- | --- | --- | --- | --- | --- | --- | --- | --- | --- | --- |
|  | *Estimates* | *std. Error* | *Estimates* | *std. Error* | *Estimates* | *std. Error* | *Estimates* | *std. Error* | *Estimates* | *std. Error* | *Estimates* | *std. Error* |
| **Intercept** | -0.02 | 0.07 | 0.04 | 0.08 | -0.05 | 0.08 | -0.04 | 0.07 | -0.05 | 0.06 | -0.10 | 0.05 |
| **Age** |  |  |  |  |  |  |  |  |  |  |  |  |
| 25-44 |  |  | 0.06 | 0.04 | 0.06 | 0.04 | 0.05 | 0.04 | 0.05 | 0.04 | 0.08 ^*^ | 0.04 |
| 45-64 |  |  | 0.17 ^***^ | 0.05 | 0.16 ^***^ | 0.05 | 0.15 ^**^ | 0.05 | 0.15 ^***^ | 0.05 | 0.20 ^***^ | 0.04 |
| More than 64 |  |  | 0.35 ^***^ | 0.07 | 0.36 ^***^ | 0.07 | 0.34 ^***^ | 0.07 | 0.32 ^***^ | 0.07 | 0.42 ^***^ | 0.07 |
| **Female dummy** |  |  | 0.01 | 0.03 | 0.00 | 0.03 | 0.00 | 0.03 | 0.00 | 0.03 | 0.02 | 0.03 |
| **Culture dummy** |  |  | -0.28 ^**^ | 0.10 | -0.26 ^**^ | 0.09 | -0.26 ^**^ | 0.08 | -0.24 ^***^ | 0.06 | -0.22 ^***^ | 0.06 |
| **Goal importance** |  |  |  |  | 0.21 ^***^ | 0.02 | 0.20 ^***^ | 0.02 | 0.18 ^***^ | 0.02 | 0.13 ^***^ | 0.02 |
| **Openness to experience** |  |  |  |  |  |  | 0.09 ^***^ | 0.02 | 0.09 ^***^ | 0.02 | 0.03 ^*^ | 0.02 |
| **Valence of music** |  |  |  |  |  |  |  |  | -0.11 ^***^ | 0.02 | -0.10 ^***^ | 0.02 |
| **Music-induced nostalgia** |  |  |  |  |  |  |  |  | 0.12 ^***^ | 0.02 | 0.04 ^**^ | 0.02 |
| **Music's importance** |  |  |  |  |  |  |  |  |  |  | 0.37 ^***^ | 0.02 |
|  |  |  |  |  |  |  |  |  |  |  |  |  |
| **Random Effects** |  |  |  |  |  |  |  |  |  |  |  |  |
| σ^2^ | 0.95 |  | 0.95 |  | 0.93 |  | 0.93 |  | 0.91 |  | 0.79 |  |
| τ_00 country_ | 0.04 |  | 0.03 |  | 0.02 |  | 0.02 |  | 0.01 |  | 0.01 |  |
| ICC _country_ | 0.04 |  |  |  |  |  |  |  |  |  |  |  |
| N _country_ | 11 |  | 11 |  | 11 |  | 11 |  | 11 |  | 11 |  |
| Observations | 3865 |  | 3865 |  | 3865 |  | 3865 |  | 3865 |  | 3865 |  |
| Marginal R^2^ / Conditional R^2^ | 0.000 / 0.045 |  | 0.025 / 0.050 |  | 0.042 / 0.062 |  | 0.051 / 0.067 |  | 0.074 / 0.083 |  | 0.195 / 0.202 |  |

Note: Culture dummy = 1 for individualistic culture, 0 for collectivistic culture. Reference age is less than 24.

Female dummy = 1 for female, 0 for males.

* *p*<0.05   ** *p*<0.01   *** *p*<0.001

**Table S8.** Overview of main statistical outcomes of multilevel regression models testing which variables predict the rated efficiency of music in *‘*Connecting with myself and detachment from the surroundings’.

|  | **Model-0** | | **Model-1** | | **Model-2** | | **Model-3** | | **Model-4** | | **Model-5** | |
| --- | --- | --- | --- | --- | --- | --- | --- | --- | --- | --- | --- | --- |
|  | *Estimates* | *std. Error* | *Estimates* | *std. Error* | *Estimates* | *std. Error* | *Estimates* | *std. Error* | *Estimates* | *std. Error* | *Estimates* | *std. Error* |
| (Intercept) | 0.00 | 0.06 | 0.06 | 0.10 | -0.03 | 0.10 | -0.02 | 0.09 | -0.03 | 0.08 | -0.08 | 0.05 |
| **Age** |  |  |  |  |  |  |  |  |  |  |  |  |
| 25-44 |  |  | -0.05 | 0.04 | -0.07 | 0.04 | -0.07 | 0.04 | -0.07 | 0.04 | -0.01 | 0.04 |
| 45-64 |  |  | -0.11 ^*^ | 0.05 | -0.11 ^*^ | 0.05 | -0.14 ^**^ | 0.05 | -0.13 ^**^ | 0.05 | -0.05 | 0.04 |
| More than 64 |  |  | -0.13 | 0.08 | -0.12 | 0.08 | -0.13 | 0.08 | -0.14 | 0.08 | 0.04 | 0.07 |
| **Female dummy** |  |  | 0.02 | 0.03 | 0.01 | 0.03 | 0.01 | 0.03 | 0.01 | 0.03 | 0.02 | 0.03 |
| **Culture dummy** |  |  | -0.03 | 0.13 | -0.00 | 0.13 | -0.00 | 0.11 | 0.01 | 0.10 | -0.01 | 0.06 |
| **Goal importance** |  |  |  |  | 0.18 ^***^ | 0.02 | 0.17 ^***^ | 0.02 | 0.15 ^***^ | 0.02 | 0.09 ^***^ | 0.02 |
| **Openness to experience** |  |  |  |  |  |  | 0.11 ^***^ | 0.02 | 0.11 ^***^ | 0.02 | 0.05 ^**^ | 0.01 |
| **Valence of music** |  |  |  |  |  |  |  |  | -0.09 ^***^ | 0.02 | -0.08 ^***^ | 0.01 |
| **Music-induced nostalgia** |  |  |  |  |  |  |  |  | 0.12 ^***^ | 0.02 | 0.03 | 0.02 |
| **Music's importance** |  |  |  |  |  |  |  |  |  |  | 0.48 ^***^ | 0.02 |
|  |  |  |  |  |  |  |  |  |  |  |  |  |
| **Random Effects** |  |  |  |  |  |  |  |  |  |  |  |  |
| σ^2^ | 0.95 |  | 0.95 |  | 0.94 |  | 0.93 |  | 0.91 |  | 0.74 |  |
| τ_00_ _country_ | 0.03 |  | 0.04 |  | 0.04 |  | 0.03 |  | 0.02 |  | 0.01 |  |
| ICC _country_ | 0.04 |  |  |  |  |  |  |  |  |  |  |  |
| N _country_ | 11 |  | 11 |  | 11 |  | 11 |  | 11 |  | 11 |  |
| Observations | 3793 |  | 3793 |  | 3793 |  | 3793 |  | 3793 |  | 3793 |  |
| Marginal R^2^ / Conditional R^2^ | 0.000 / 0.035 |  | 0.002 / 0.044 |  | 0.015 / 0.054 |  | 0.028 / 0.058 |  | 0.047 / 0.071 |  | 0.242 / 0.250 |  |

Note: Culture dummy = 1 for individualistic culture, 0 for collectivistic culture. Reference age is less than 24.

Female dummy = 1 for female, 0 for males.

* *p*<0.05   ** *p*<0.01   *** *p*<0.001

**Table S9.** Overview of main statistical outcomes of multilevel regression models testing the effects of Age and Culture on rated efficiency of music in obtaining *‘*Enjoyment and maintaining good mood'.

|  | **Model-0** | | **Model-1** | | **Model-2** | | **Model-3** | |
| --- | --- | --- | --- | --- | --- | --- | --- | --- |
| ***Predictors*** | ***Estimates*** | ***std. Error*** | ***Estimates*** | ***std. Error*** | ***Estimates*** | ***std. Error*** | ***Estimates*** | ***std. Error*** |
| **Intercept** | 6.13 ^***^ | 0.07 | 6.23 ^***^ | 0.07 | 6.27 ^***^ | 0.11 | 6.29 ^***^ | 0.11 |
| **Age** |  |  |  |  |  |  |  |  |
| 25-44 |  |  | -0.12 ^**^ | 0.04 | -0.12 ^**^ | 0.04 | -0.16 ^**^ | 0.05 |
| 45-64 |  |  | -0.15 ^***^ | 0.04 | -0.15 ^***^ | 0.04 | -0.19 ^**^ | 0.06 |
| More than 64 |  |  | -0.18 ^*^ | 0.07 | -0.18 ^*^ | 0.07 | -0.14 | 0.12 |
| **Culture dummy** |  |  |  |  | -0.08 | 0.15 | -0.14 | 0.16 |
| **Age × Culture** |  |  |  |  |  |  |  |  |
| 25-44 **×** Culture |  |  |  |  |  |  | 0.08 | 0.08 |
| 45-64 **×** Culture |  |  |  |  |  |  | 0.09 | 0.09 |
| 65+ **×** Culture |  |  |  |  |  |  | -0.03 | 0.15 |
|  |  |  |  |  |  |  |  |  |
| **Random Effects** |  |  |  |  |  |  |  |  |
| σ^2^ | 1.09 |  | 1.08 |  | 1.08 |  | 1.08 |  |
| τ_00_ _country_ | 0.05 |  | 0.05 |  | 0.05 |  | 0.06 |  |
| ICC _country_ | 0.04 |  | 0.04 |  | 0.05 |  | 0.05 |  |
| N _country_ | 11 |  | 11 |  | 11 |  | 11 |  |
| Observations | 4872 |  | 4872 |  | 4872 |  | 4872 |  |
| Marginal R^2^ / Conditional R^2^ | 0.000 / 0.041 |  | 0.003 / 0.048 |  | 0.005 / 0.053 |  | 0.005 / 0.054 |  |

Note: Culture dummy = 1 for individualistic culture, 0 for collectivistic culture. Reference age is less than 24.

Female dummy = 1 for female, 0 for males.

* *p*<0.05   ** *p*<0.01   *** *p*<0.001
